# Combined mutation of *Vhl* and *Trp53* causes renal cysts and tumours in mice

**DOI:** 10.1002/emmm.201202231

**Published:** 2013-04-22

**Authors:** Joachim Albers, Michal Rajski, Désirée Schönenberger, Sabine Harlander, Peter Schraml, Adriana von Teichman, Strahil Georgiev, Peter J Wild, Holger Moch, Wilhelm Krek, Ian J Frew

**Affiliations:** 1 Institute of Physiology, University of ZurichZurich, Switzerland; 2 Competence Center for Systems Physiology and Metabolic Diseases, ETH Zurich and University of ZurichZurich, Switzerland; 3 Zurich Center for Integrative Human Physiology, University of ZurichZurich, Switzerland; 4 Institute of Surgical Pathology, University Hospital ZurichZurich, Switzerland; 5 Institute of Molecular Health SciencesETH Zurich, Zurich, Switzerland

**Keywords:** ccRCC, cyst, p53, *VHL*

## Abstract

The combinations of genetic alterations that cooperate with von Hippel–Lindau (*VHL*) mutation to cause clear cell renal cell carcinoma (ccRCC) remain poorly understood. We show that the *TP53* tumour suppressor gene is mutated in approximately 9% of human ccRCCs. Combined deletion of *Vhl* and *Trp53* in primary mouse embryo fibroblasts causes proliferative dysregulation and high rates of aneuploidy. Deletion of these genes in the epithelium of the kidney induces the formation of simple cysts, atypical cysts and neoplasms, and deletion in the epithelia of the genital urinary tract leads to dysplasia and tumour formation. Kidney cysts display a reduced frequency of primary cilia and atypical cysts and neoplasms exhibit a pro-proliferative signature including activation of mTORC1 and high expression of Myc, mimicking several cellular and molecular alterations seen in human ccRCC and its precursor lesions. As the majority of ccRCC is associated with functional inactivation of *VHL*, our findings suggest that for a subset of ccRCC, loss of p53 function represents a critical event in tumour development.

## INTRODUCTION

Clear cell renal cell carcinoma (ccRCC) accounts for approximately 80% of kidney tumours and thereby approximately 2.5% of all types of human malignancy. The von Hippel–Lindau (*VHL*) tumour suppressor gene is mutated, deleted or epigenetically silenced in up to 85% of all sporadic cases of ccRCC (Maher, 2013). Germline inheritance of a single mutant allele of *VHL* gives rise to the dominantly inherited VHL familial cancer syndrome which predisposes not only to the formation of ccRCC, but also to cystic lesions in the kidney and pancreas as well as to diverse types of tumours in the central nervous system, eye, ear, pancreas, adrenal gland, epididymis and broad ligament (Kaelin, 2002).

The pVHL protein has been ascribed several distinct biochemical activities and implicated in the regulation of diverse cellular processes, dysregulation of any or all of which could be envisaged to play important roles in tumour formation (Frew & Krek, 2007). Two lines of evidence however suggest that loss of pVHL function alone is insufficient for tumour initiation in the kidney. Kidneys of patients with an inherited *VHL* mutation frequently display cystic lesions as well as ccRCC. Since some pVHL-deficient proliferative cysts contain micro-foci of ccRCC, it is believed that, at least in some cases, cysts represent a precursor lesion in the evolution of malignant ccRCC (Lubensky et al, 1996; Walther et al, 1995). Detailed analysis of regions of normal histology in these kidneys revealed that VHL patient kidneys likely contain many thousands of individual isolated cells that are null for pVHL function (Mandriota et al, 2002; Montani et al, 2010). pVHL-deficient cysts and ccRCC apparently arise infrequently in comparison to the total frequency of *VHL* mutation. Secondly, heterozygous deletion of the mouse homologue of the *VHL* gene, *Vhl* (previously referred to as *Vhlh*), in the entire mouse (Haase et al, 2001), or homozygous deletion under the control of kidney-specific Cre transgenes, does not lead to proliferative dysregulation or tumour formation in the kidney (Frew et al, 2008b; Rankin et al, 2006). Multiple genetic mutations appear to be required to cause proliferation and transformation of pVHL-deficient cells.

Genes that are mutated at high frequency in diverse human epithelial tumours, including *PTEN*, *EGFR*, *ERBB2*, *BRAF*, *RAS* family genes, *RB1* and *APC*, are either not mutated or are mutated at relatively low frequencies (<10%) in ccRCC. Rather, ccRCC frequently (41%) harbour mutations in the chromatin remodelling gene *PBRM1* (Varela et al, 2011) and in several genes involved in histone modification (Dalgliesh et al, 2010) and protein ubiquitination and de-ubiquitination (Guo et al, 2012; Pena-Llopis et al, 2012). Several chromosomal regions are frequently amplified or deleted and numerous genes are frequently hypermethylated in ccRCC (Maher, 2013), implying that there may be many different combinations of genetic alterations that can cooperate with loss of *VHL* function to cause tumour formation. Our previous studies demonstrate that low-frequency mutations could be functionally important in ccRCC formation; co-deletion of *Vhl* and *Pten* in the mouse kidney led to the formation of proliferative cysts, mimicking the precursor lesions of ccRCC that arise in human VHL patients (Frew et al, 2008b). Several studies, including data presented herein, have shown that *TP53* is mutated in a subset of ccRCC (http://cancer.sanger.ac.uk/cosmic). We demonstrate that combined mutation of *Vhl* and *Trp53* causes dysregulation of cellular proliferation in primary mouse embryo fibroblasts (MEFs) and kidney epithelial cells and results in the formation of kidney cysts and neoplastic lesions in kidneys as well as tumours in genital tract organs.

## RESULTS

### *TP53* mutations occur in sporadic ccRCCs

We sequenced the entire *VHL* gene and exons 5–8 of the *TP53* gene in 54 cases of sporadic ccRCC (Table 1). As expected, missense or truncating *VHL* mutations were observed in 73% of the tumours. Immunohistochemistry for the HIF1α-inducible proteins CA9 and Glut1, and for HIF1α itself, revealed moderate or strong expression of at least one of these markers in all but two of the tumours, verifying the well-described hypoxic signature associated with loss of function of pVHL. *TP53* mutations that affected the coding region were detected in 5 (9%) tumours, all of which are either previously described pathogenic mutations or are predicted to be pathogenic. One tumour harboured both *VHL* and *TP53* mutations, while the other four *TP53* mutant tumours were wild-type for *VHL*. While methylation analyses for the *VHL* gene were not possible in these samples, it is likely that pVHL expression may be silenced in these tumours as they showed very high immunohistochemical staining for the HIFα target genes. In agreement with our data, the COSMIC database (http://cancer.sanger.ac.uk/cosmic) lists 30 of 209 (14.4%) tumours that display *TP53* coding region mutations. Unfortunately the *VHL* mutation status of these tumours is in most cases unknown. Thus, *TP53* is mutated in a significant fraction of sporadic ccRCCs.

**Table 1:** *VHL* and *TP53* mutations and CA9, GLUT1 and HIF1α immunohistochemistry in sporadic cases of human ccRCC

pT	*VHL* sequencing			Immunohistochemistry			*TP53* sequencing			
			
	Exon 1	Exon 2	Exon 3	CA9	Glut1	HIF1α	Exon 5	Exon 6	Exon 7	Exon 8
3	–	–	A207CfsX49	2	0	0	–	–	–	–
3b	–	T124RfsX5	–	2	2	1	–	–	–	–
3	–	H115SfsX17	–	2	1	0	–	–	–	–
3b	N78S	–	–	2	2	1	n.a.	–	–	–
3b	–	–	–	1	2	0	K139K	–	–	–
3a	–	V155CfsX4	–	2	0	2	–	–	–	–
3b	–	–	–	1	0	n.a.	Q165X	–	–	–
3	–	–	–	2	1	1	n.a.	–	–	–
3b	–	–	R167_V170del	2	2	2	–	–	–	–
3a	–	–	–	1	1	1	–	–	–	–
3b	P99QfsX60	–	–	2	2	1	–	R213R	–	–
3b	S68T	–	–	2	2	0	–	–	–	–
3a	–	–	–	0	1	0	–	–	–	–
3	S65T	–	–	2	2	0	–	–	–	–
3a	–	–	I180V	2	2	2	–	–	–	–
3	–	V130F	–	2	1	0	–	–	L257L	–
3b	–	L153TfsX21	–	2	1	1	–	R142R	–	–
4	–	–	V170D	2	2	1	–	–	–	–
3a	–	–	L158V	2	n.a.	0	–	–	–	–
3a	–	–	H191H	2	2	0	–	P219L	–	–
3a	–	–	–	2	2	0	H179L	–	–	–
3a	–	W117R	–	2	2	2	–	–	–	–
3b	–	–	R161P	0	0	1	–	–	–	–
4	Y98X	–	–	0	2	1	–	–	–	–
3a	T100SfsX59	–	–	2	2	2	–	–	–	–
4	–	–	–	0	2	n.a.	–	–	–	–
3a	–	–	–	2	2	0	–	–	–	–
3	L101P	–	–	2	2	0	–	–	–	–
3a	–	–	–	2	2	1	–	–	–	–
3a	Y112D	–	–	n.a.	n.a.	0	–	–	–	–
3b	D92AfsX36	–	–	2	2	0	–	–	–	–
3b	V62CfsX5	–	–	1	n.a.	0	–	–	–	–
3	–	–	–	1	2	0	–	–	–	–
3b	c.340 + 1G>T	–	–	2	2	0	–	–	–	–
3	S65L	–	–	2	2	1	–	–	–	–
3	–	c.341-2A>G	–	2	2	0	–	–	–	–
3a	–	–	–	2	2	1	–	–	–	–
4	S68X	–	–	2	2	0	–	–	–	–
3b	Y98N	–	–	2	1	0	–	R213R	–	–
3b	S68X	–	–	2	2	0	–	–	–	–
3b	R107VfsX45	–	–	2	2	1	–	–	–	–
3b	Q73X	–	–	0	2	0	–	–	–	R273C
3a	–	–	R161X	2	n.a.	1	–	–	–	–
3b	–	–	V181KfsX14	n.a.	n.a.	0	–	–	–	–
3	n.d.	n.d.	n.d.	2	2	0	–	–	–	–
3	n.d.	n.d.	n.d.	2	2	0	–	–	–	–
3	n.d.	n.d.	n.d.	2	2	1	–	–	–	–
3	n.d.	n.d.	n.d.	0	1	1	–	–	–	–
3	n.d.	n.d.	n.d.	2	2	2	–	–	–	–
3	n.d.	n.d.	n.d.	0	1	1	–	–	–	–
2	n.d.	n.d.	n.d.	2	1	1	–	–	–	–
4	n.d.	n.d.	n.d.	2	2	0	–	1bp ins^*^	–	–
3	n.d.	n.d.	n.d.	1	2	1	–	–	–	–
3	n.d.	n.d.	n.d.	2	1	1	–	–	–	–

Grey shading highlights a mutation that causes a coding alteration. Amino acid alterations are shown by single letter code, del = deletion, fs = frame shift, X = new stop codon, n.a. = not analysable, n.d. = not determined, ^*^ = bp insertion not identifiable, 0 = no staining, 1 = moderate staining, 2 = strong staining.

### *Trp53* mutation allows immortalisation of *Vhl* mutant primary mouse embryo fibroblasts

**Figure 1:**
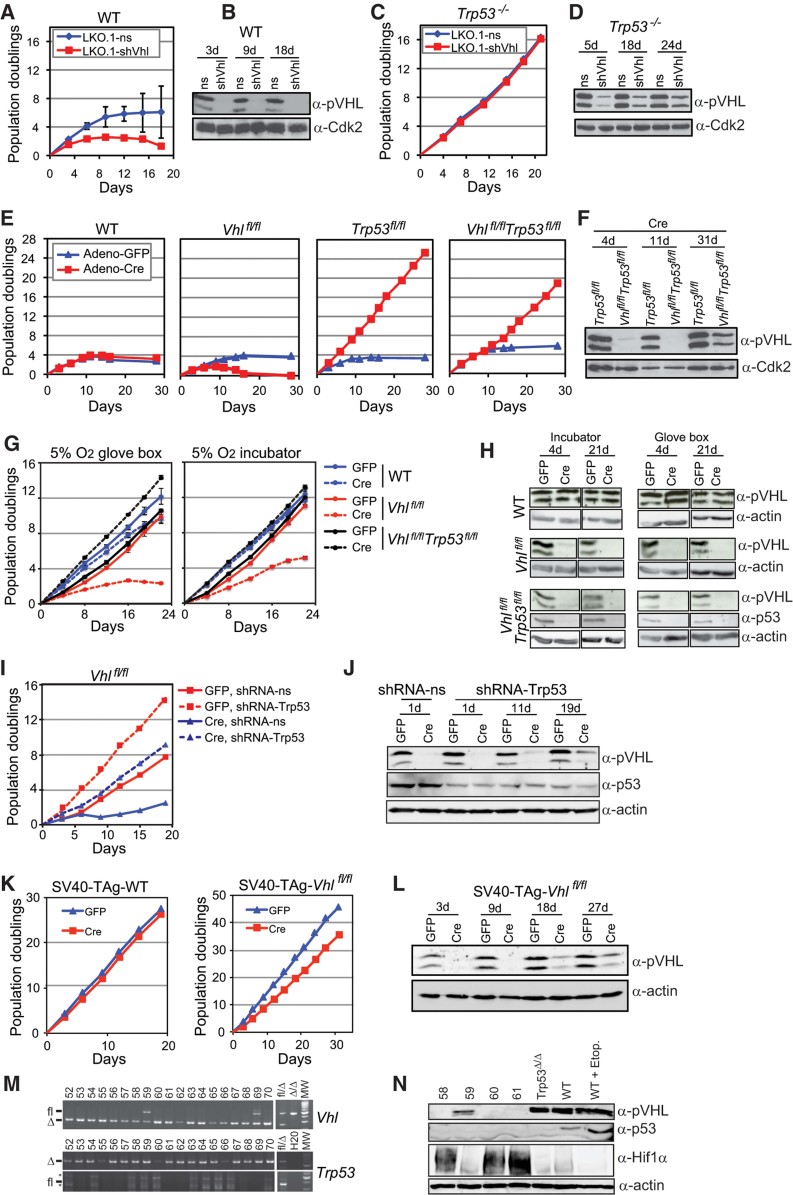
*Trp53* mutation allows immortalized proliferation of *Vhl* null MEFs **A,C.** Proliferation assays of wild-type (A) and *Trp53*^*−/−*^ (C) MEFs following infection with pLKO.1 lentiviruses expressing a non-silencing sequence (ns) or shRNA directed against *Vhl* (shVhl).**B,D.** Western blotting analysis for pVHL in cells from A and C at the time points (number of days after infection) indicated. Immunoblotting using an antibody against Cdk2 served as a loading and transfer control.**E.** Proliferation assays of wild-type, *Vhl*^*fl/fl*^, *Trp53*^*fl/fl*^ or *Vhl*^*fl/fl*^*Trp53*^*fl/fl*^ MEFs following infection with adenoviruses expressing GFP (GFP) or Cre-GFP (Cre).**F.** Western blotting analysis of *Trp53*^*fl/fl*^ and *Vhl*^*fl/fl*^*Trp53*^*fl/fl*^ MEFs in E at the indicated time points. Immunoblotting using an antibody against actin served as a loading and transfer control.**G.** Proliferation assays of wild-type, *Vhl*^*fl/fl*^ and *Vhl*^*fl/fl*^*Trp53*^*fl/fl*^ MEFs in 5% oxygen.**H.** Western blotting analysis of cells from G at the indicated timepoints.**I.** Proliferation assays of *Vhl*^*fl/fl*^ MEFs infected with GFP or Cre and lentiviruses expressing an empty miR30 shRNA (shRNA-ns) or miR30-format shRNA directed against *Trp53* (shRNA-Trp53).**J.** Western blotting analysis of cells from I at the indicated time points.**K.** Proliferation assays of SV40 T-antigen transformed WT and *Vhl*^*fl/fl*^ MEFs following GFP or Cre infection.**L.** Western blotting analysis of SV40-TAg-*Vhl*^*fl/fl*^ from K at the indicated timepoints.**M.** Cell lines (52–70) derived from Cre-infected *Vhl*^*fl/fl*^*Trp53*^*fl/fl*^ MEFs genotyped for floxed (fl) and deleted (Δ) *Vhl* and *Trp53* alleles. Samples with known *Vhl* and *Trp53* genotypes served as controls, MW: molecular weight markers, * non-specific bands.**N.** Western blotting analysis of clones 58–61 for pVHL, p53 and Hif1α, confirming the loss of p53 expression and presence and functionality of pVHL expression in clone 59. Lysates from *Trp53* null MEFs, wild-type MEFs or wild-type MEFs treated with etoposide (10 µM, 6 h) served as controls for the p53 protein.

We first utilized primary MEFs to investigate potential cooperative interactions of the mouse *Vhl* and *Trp53* genes in proliferative control. Deletion of *Vhl* in primary and transformed MEFs induces proliferation arrest and/or the onset of premature senescence (Mack et al, 2005; Welford et al, 2010; Young et al, 2008). Consistent with these findings, lentiviral-mediated shRNA knockdown of *Vhl* led to rapid loss of proliferative capacity of wild-type MEFs (Fig 1A). In contrast, *Vhl* knockdown had no anti-proliferative effect on *Trp53*^*−/−*^ MEFs (Fig 1C). Interestingly, while efficient reduction of pVHL protein was maintained throughout the duration of the experiment in wild-type cells (Fig 1B), the extent of knockdown rapidly diminished with increasing passage in *Trp53*^*−/−*^ cells (Fig 1D), suggesting that there is a proliferative selection for those cells in which the knockdown occurs less efficiently. To attempt to overcome this problem of selection, MEFs from wild-type, *Vhl*^*fl/fl*^, *Trp53*^*fl/fl*^ and *Vhl*^*fl/fl*^*Trp53*^*fl/fl*^ mice were infected with adenovirus expressing GFP (Adeno-GFP) as control or with adenovirus expressing a Cre-GFP fusion protein (Adeno-Cre) to delete the floxed genes. *Vhl* knockout MEFs rapidly lost proliferative capacity whereas *Vhl/Trp53* double deletion allowed proliferation, albeit at a slower rate of proliferation than *Trp53* deletion alone (Fig 1E). Consistent with a rescue of senescence, *Vhl/Trp53* double null cells retained the appearance of proliferating cells while *Vhl* deletion caused an enlarged, flattened cell morphology characteristic of senescence (Supporting Information Fig 1). None of the gene deletions allowed the formation of colonies in soft agar, demonstrating that the cells are immortalized but not transformed. Western blotting 4 days after infection confirmed the high efficiency of gene deletion but after approximately 20–30 days of continuous proliferation, a significant restoration of pVHL expression was evident in the *Vhl*^*fl/fl*^*Trp53*^*fl/fl*^ Cre-infected cell population (Fig 1F), suggesting a mixed cell population in which *Vhl* null cells have a proliferative disadvantage in comparison to an initially rare population of pVHL-expressing cells (see results below).

In light of recent findings showing that senescence induced by loss of *Vhl* can be rescued by culturing cells at 2 or 5% oxygen (Welford et al, 2010), we conducted a series of experiments in which cells were grown at 5% oxygen. We observed a reproducible but only partial restoration of proliferative capacity following *Vhl* knockout (Fig 1G) when cells were cultured either in a glove box incubator to ensure constant oxygen tension throughout the experiment or in a conventional oxygen incubator where cells were briefly exposed to 21% oxygen every 3 days during passaging. In either incubator, wild-type MEFs did not enter senescence within 50 days of culture at 5% oxygen but typically entered senescence after about 10–12 days at 21% oxygen, verifying our culture system against published data (Parrinello et al, 2003). In light of this partial rescue of proliferation of *Vhl* mutant cells, all subsequent proliferation experiments were conducted at 5% oxygen in a conventional incubator.

To permit comparison of proliferation rates in an isogenic background, we knocked down *Trp53* in *Vhl*^*fl/fl*^ MEFs. *Trp53* knockdown rescued the proliferation defect of *Vhl* knockout (Adeno-Cre treated) cells but these cells proliferated more slowly than control (Adeno-GFP treated) cells with *Trp53* knockdown alone (Fig 1I). The *Vhl* knockout/*Trp53* knockdown cultures became enriched with pVHL-expressing cells over time (Fig 1J). A similar reduction in proliferation rate (Fig 1K) and passage-dependent enrichment of pVHL-expressing cells in the cell population (Fig 1L) was also observed in cultures where *Vhl* was deleted from *Vhl*^*fl/fl*^ MEFs that had been transformed with SV40 Large T-Antigen to simultaneously inactivate both the p53 and pRB-dependent cell cycle checkpoints. Thus, loss of pVHL compromises cellular proliferation in MEFs in a manner independent of the p53 and pRB cell cycle checkpoints.

Given the strong selection against pVHL-expressing cells in bulk population experiments, we performed experiments using single cells to definitively address the question of whether *Vhl*/*Trp53* double null cells are truly immortalized. While wild-type MEFs undergo cellular senescence when plated as single cells, *Trp53* null cells form colonies allowing the generation of immortalized cell lines founded from single cells. Two days after infection of *Vhl*^*fl/fl*^*Trp53*^*fl/fl*^ primary MEFs with Adeno-Cre, cells were plated at a density of 0.5 cells/well in six 96-well plates. Cell lines were generated over a period of 6 weeks and genotyped to detect the floxed or deleted *Vhl* allele, allowing a retrospective assessment of the genotype of the initiating cell of the cell line. From a theoretical maximum of 288 cell lines, 135 cell lines were generated. One hundred and thirty-three of these harboured homozygous deletion of *Vhl*, while two were heterozygous for the floxed and deleted allele (Fig 1M). All cell lines showed homozygous deletion of the floxed *Trp53* gene (Fig 1M). Western blotting of a subset of these cell lines confirmed the PCR genotyping results (Fig 1N). Thus, *Trp53* deletion efficiently allows immortalization of *Vhl* null MEFs. It is likely that the rare cells in which only one floxed *Vhl* allele (but both floxed *Trp53* alleles) has undergone Cre-mediated recombination have a proliferative advantage over the *Vhl/Trp53* null cells, allowing them to accumulate over time in bulk populations.

### *Trp53* mutation rescues proliferation of *Vhl* mutant primary renal epithelial cells

**Figure 2:**
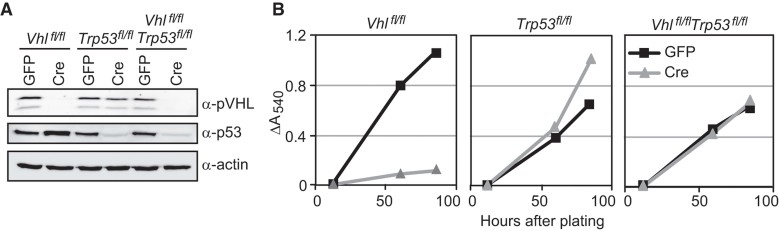
*Trp53* deletion rescues proliferative defects of *Vhl* null primary kidney epithelial cells Western blotting analysis of primary kidney epithelial cell cultures derived from *Vhl*^*fl/fl*^, *Trp53*^*fl/fl*^ or *Vhl*^*fl/fl*^*Trp53*^*fl/fl*^ mice 3 days after infection with adenoviruses expressing GFP or Cre.Proliferation of cells from A assessed using an SRB assay to detect increase in total protein content of the culture over time.

To investigate the cooperative effects of combined *Vhl* and *Trp53* deletion in a disease-relevant cell type we cultured primary mouse renal epithelial cells from the various floxed mouse strains at 5% oxygen and deleted *Vhl* and/or *Trp53* using Adeno-Cre or using Adeno-GFP as control (Fig 2A). While long-term assays of renal epithelial cell behaviour are not possible due to the epithelial to mesenchymal transition that occurs over time, in short term assays we observed that deletion of *Vhl* inhibited the proliferation of renal epithelial cells and co-deletion of *Trp53* rescued this inhibition of proliferation (Fig 2B). Unlike in MEFs, cultures of *Trp53* or *Vhl/Trp53* null renal epithelial cells formed colonies when plated at single cell density with very low efficiency (<1%), and did not grow in soft agar, demonstrating that these cells are not immortalized or transformed.

### Enhanced rates of aneuploidy in *Trp53* and *Vhl/Trp53* mutant MEFs

**Figure 3:**
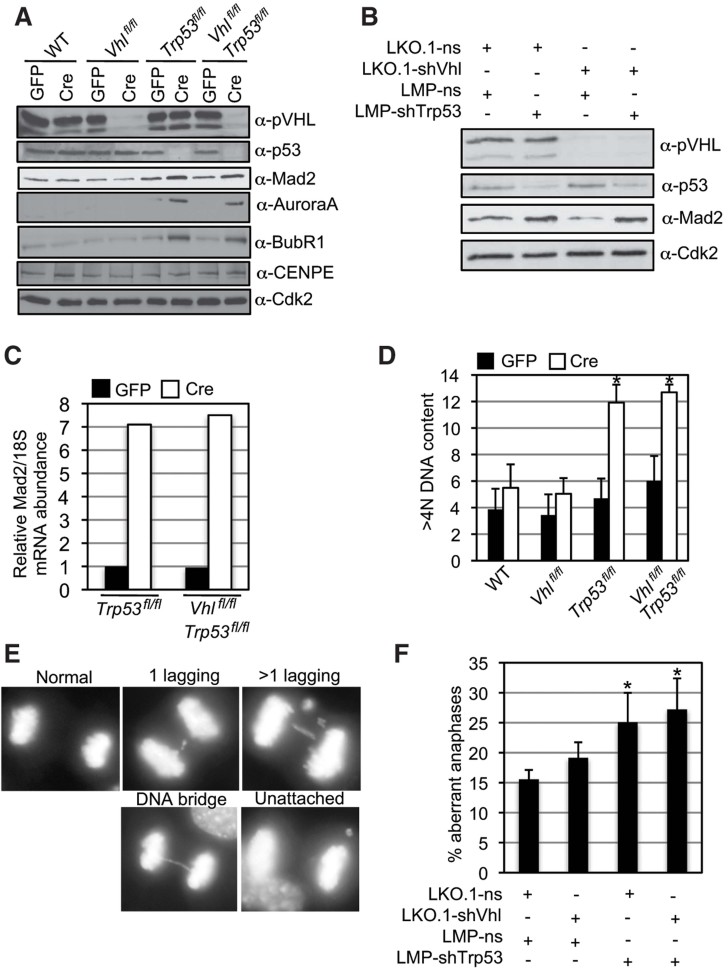
High rates of aneuploidy in *Trp53* and *Vhl*/*Trp53* null MEFs Western blotting analysis of wild-type, *Vhl*^*fl/fl*^, *Trp53*^*fl/fl*^ or *Vhl*^*fl/fl*^*Trp53*^*fl/fl*^ MEFs infected with adenoviruses expressing GFP (GFP) or Cre-GFP (Cre).Western blotting analysis of wild-type MEFs infected with combinations of pLKO.1 lentiviruses expressing a non-silencing sequence (ns) or shRNA directed against *Vhl* (shVhl) and LMP retroviruses expressing a non-silencing sequence (ns) or shRNA directed against *Trp53* (shTrp53).Real-time quantitative PCR analysis of Mad2 mRNA abundance normalized to 18S mRNA abundance in GFP and Cre infected *Trp53*^*fl/fl*^ and *Vhl*^*fl/fl*^*Trp53*^*fl/fl*^ MEFs.Frequency of cells with greater than 4N DNA content as assessed by flow cytometric analysis. Results represent mean ± SD of triplicate samples and * represents statistically significant differences between GFP and Cre treated cells of the same genotype (Student's *t*-test, *p* < 0.01).Depiction of a normal anaphase and examples of anaphases showing a range of chromosome segregation defects.Anaphases of cells from B were scored five days after infection according to the scheme shown in E and the percentage of aberrant anaphases calculated. Data represent mean ± SD of triplicate samples (in each *n* > 120 anaphases were counted) and * represents statistically significant differences to cells infected with both ns vectors (Student's *t*-test, *p* < 0.01).

One driving force in the evolution of tumours is aneuploidy. Loss of pVHL, through an uncharacterized mechanism, results in lower levels of the mitotic checkpoint protein Mad2, which alone causes a moderate elevation of levels of aneuploidy but when combined with reduction in expression of another mitotic spindle checkpoint protein, CENP-E, induces a dramatic increase in aneuploidy (Thoma et al, 2009). Here we confirm that deletion (Fig 3A) or knockdown (Fig 3B) of *Vhl* causes a reduction of Mad2 protein expression. Loss of *Trp53* function in MEFs also causes higher levels of aneuploidy and polyploidy and has been shown to result in aberrantly elevated expression levels of the checkpoint proteins Aurora A (Mao et al, 2007), Mad2 and separase (Pati et al, 2004). Western blotting of *Trp53*^*fl/fl*^ cells infected with Adeno-Cre (Fig 3A) revealed elevated expression levels of Aurora A and Mad2, as well as elevated expression of BubR1, another spindle checkpoint protein, but no change in the expression levels of CENP-E. To our knowledge this is the first report of this effect of p53 on BubR1. Since double mutation of *Mad2* and *Trp53* has been shown to lead to dramatic levels of aneuploidy (Burds et al, 2005), we investigated whether the combined effects of loss of *Vhl* and *Trp53* on the expression of various mitotic spindle checkpoint proteins would have a similar effect. However, *Vhl/Trp53* double knockout (Fig 3A) or double knockdown (Fig 3B) cells displayed higher than normal levels of Mad2. This was presumably due to the elevation in mRNA abundance of *Mad2* in *Trp53* and *Vhl/Trp53* knockout MEFs (Fig 3C), consistent with previous observations that p53 represses *Mad2* mRNA expression (Pati et al, 2004), overriding the effect of loss of *Vhl* in reducing Mad2 expression. Thus, in terms of the expression of several proteins whose levels regulate spindle checkpoint function, *Vhl/Trp53* double null cells are similar to *Trp53* null cells. Functional studies supported this idea. Flow cytometry revealed that cultures of *Trp53* null MEFs accumulated polyploid cells at the same frequency as cultures of *Vhl/Trp53* double null MEFs (Fig 3D). To directly monitor the integrity of the mitotic spindle checkpoint in an isogenic background we performed lentiviral-mediated knockdown of *Vhl* and/or *Trp53* in MEFs and performed fluorescence microscopy to detect aberrant anaphases that are characterized by the presence of lagging or unattached chromosomes or DNA bridges (Fig 3E). Knockdown of *Vhl* led to a slightly increased rate of aberrant anaphases (Fig 3F), *Trp53* knockdown and *Vhl/Trp53* double knockdown both led to a statistically significant increase in the frequency of aberrant anaphases in comparison to control knockdowns, but the two genotypes were not significantly different from one another (Fig 3F).

In summary, while there appear to be no cooperative genetic effects of loss of *Vhl* and *Trp53* function on aneuploidy, *Trp53* mutation in a *Vhl* mutant background may enhance aneuploidy, which may be relevant for tumourigenesis.

### Deletion of *Vhl* and *Trp53* in mouse kidney and genital–urinary tract epithelia causes dysplasia and tumour formation

To investigate the consequences of combined deletion of *Vhl* and *Trp53* in epithelial tissues *in vivo*, *Vhl*^*fl/fl*^ and *Trp53*^*fl/fl*^ mice were interbred with *Ksp1.3-Cre* transgenic mice to generate *Ksp1.3-Cre*; *Vhl*^*fl/fl*^ (Frew et al, 2008b), *Ksp1.3-Cre*; *Trp53*^*fl/fl*^ (Wild et al, 2012) and *Ksp1.3-Cre*; *Vhl*^*fl/fl*^; *Trp53*^*fl/fl*^ mice, hereafter referred to as Vhl^Δ/Δ^, Trp53^Δ/Δ^ and Vhl^Δ/Δ^Trp53^Δ/Δ^ mice respectively. In the kidney, the *Ksp1.3-Cre* transgene induces gene deletion in the epithelial cells at the urinary pole of the glomerulus, distal tubules, loops of Henle, collecting ducts and also very infrequently in proximal tubular cells. Expression of this transgene in the Wolffian and Müllerian ducts during development also leads to gene deletion in the epithelia of the renal pelvis, ureter, vesicular glands, epididymis, vas deferens and endometrium.

Vhl^Δ/Δ^Trp53^Δ/Δ^ mice were sub-viable, with approximately 25% of mice dying within the first 3 months of life and with subsequent deaths in an apparently stochastic manner as the mice aged. Autopsy of these mice failed to reveal any obvious cause of death and no tumours were evident in any of the dead mice. This fact complicated the accrual of large cohorts of aged mice. Nonetheless, in combination with previously published analyses (Frew et al, 2008a, b) we analysed cohorts of mice at the following ages: 2–3 months (Vhl^Δ/Δ^, *n* = 8; Trp53^Δ/Δ^, *n* = 7; Vhl^Δ/Δ^Trp53^Δ/Δ^ *n* = 6), 4–8 months (Vhl^Δ/Δ^, *n* = 6; Trp53^Δ/Δ^, *n* = 10; Vhl^Δ/Δ^Trp53^Δ/Δ^ *n* = 10) and 11–13 months (Vhl^Δ/Δ^, *n* = 9; Trp53^Δ/Δ^, *n* = 10; Vhl^Δ/Δ^Trp53^Δ/Δ^ *n* = 17). Littermate mice that were negative for the *Ksp1.3-Cre* transgene served as controls for all of these cohorts.

**Figure 4:**
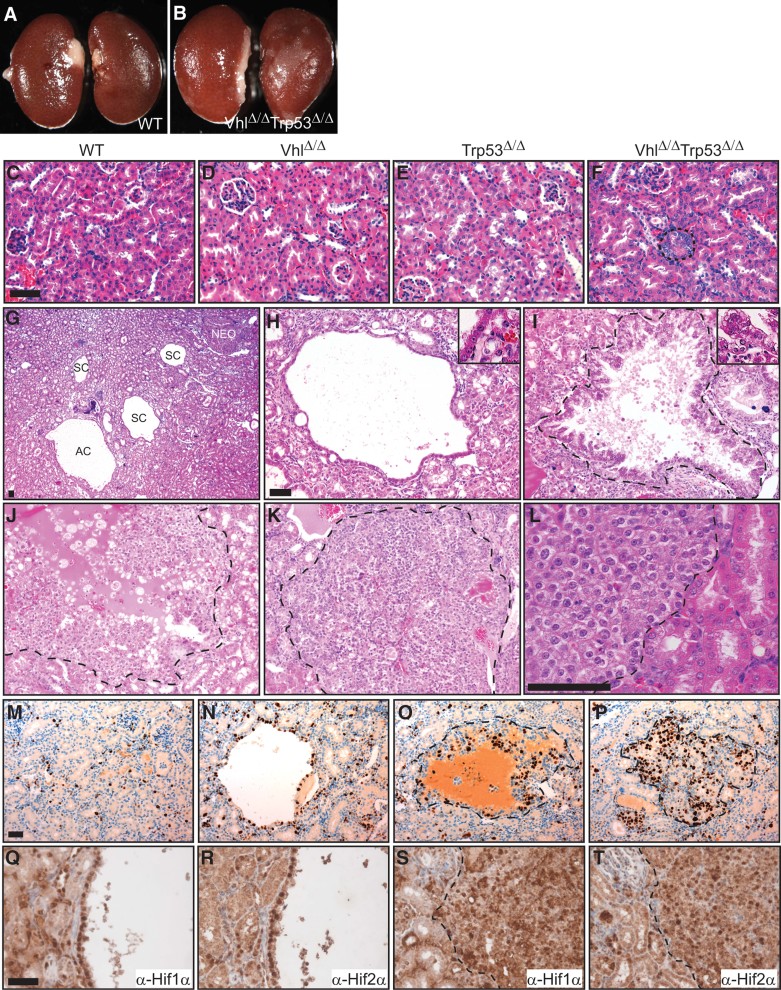
Vhl^Δ/Δ^Trp53^Δ/Δ^ mice develop kidney cysts and neoplasms C–F and Q–T are all the same magnification, H–K are the same magnification, M–P are the same magnification. Scale bars depict 50 µm. Dotted lines indicate the boundary of normal tissue and atypical cysts or neoplasms. **A,B.** Normal external appearance of kidneys from 6 month-old Vhl^Δ/Δ^Trp53^Δ/Δ^ mice.**C–F.** Histological appearance of cortex of kidneys from 6 month-old wild-type (C), Vhl^Δ/Δ^ (D), Trp53^Δ/Δ^ (E) and Vhl^Δ/Δ^Trp53^Δ/Δ^ (F) mice. The dotted region outlined in F is an example of an abnormal cluster of cells.**G.** Example of lesions arising in the cortex of a kidney from a 1-year-old Vhl^Δ/Δ^Trp53^Δ/Δ^ mouse. AC: atypical cyst, SC: simple cyst, NEO: neoplasm.**H–K.** Examples of lesions found in kidneys of one year-old Vhl^Δ/Δ^Trp53^Δ/Δ^ mice; simple tubular cyst (H), atypical cyst (I), neoplasm with cystic precursor (J) and solid neoplasm (K). Insets in H and I show high magnification of the cystic epithelium.**L.** High magnification of a solid neoplasm showing clear cell morphology and low nuclear grade.**M–P.** Representative Ki67 stainings of histologically normal epithelium (M), a simple cyst (N), an atypical cyst (O) and a neoplasm (P) in Vhl^Δ/Δ^Trp53^Δ/Δ^ mouse kidneys.**Q,R.** Anti-HIF1α and anti-HIF2α immunohistochemistry of serial sections of a simple cyst.**S,T.** Anti-HIF1α and anti-HIF2α immunohistochemistry of serial sections of a neoplastic lesion.

As previously described, kidneys of Trp53^Δ/Δ^ mice developed normally and showed no histological abnormalities within 18 months of age (Wild et al, 2012). Similarly to Vhl^Δ/Δ^ mice (Frew et al, 2008b), Vhl^Δ/Δ^Trp53^Δ/Δ^ mice developed a hydronephrosis phenotype of unknown cause but otherwise showed no defects in the structure of the nephrons at early ages. Mutation of *Trp53* in combination with *Vhl* led to a similar accumulation of nuclear HIF1α and HIF2α in tubular epithelia to that seen in *Vhl* single mutant mice (Supporting Information Fig 2). By 5 months of age small clusters of disorganized cells (Fig 4F) or micro-cysts (not shown) could infrequently be observed in the double knockout mice but not in either of the single mutant mice or control mice, suggestive of a breakdown in normal proliferative control in these cells. In comparison to the normal histological appearance of kidneys from 11- to 13-month-old control and single *Vhl* and *Trp53* mutant mice, kidneys of 13 out of 17 Vhl^Δ/Δ^Trp53^Δ/Δ^ mice aged 11–13 months mice displayed multiple hyperproliferative lesions (Fig 4G) and mild focal lymphoplasmacellular inflammation. Sections through the midline of 24 kidneys from these mice revealed 399 cysts ranging in diameter from 100 µm to 1 mm. Three hundred and forty-nine of these were lined by a single layered cuboidal epithelium (simple cyst) (Fig 4H) while 50 cysts showed multilayered micro-papillary epithelial growths projecting into the lumen (atypical cyst) (Fig 4I). Some larger cysts showed signs of regression, bleeding, cholesterol accumulation and foam cell macrophage infiltration. An additional 16 neoplastic lesions (diameter 250 µm to 1 mm) were also observed (Fig 4J and K). These lesions were non-invasive, displayed an increased mitotic index, low nuclear grade (Fig 4L) and cells grew either in a micro-papillary (Fig 4J) or solid (Fig 4K and L) growth pattern. Tumour cells typically showed weak cytoplasmic eosin staining (Fig 4L), similar to, but to a lesser extent than, the clear cell morphology seen in human ccRCC. Approximately half of the neoplasms were growing into a cystic space (Fig 4J) whereas the other lesions presented as a solid mass (Fig 4K and L). It was not possible to distinguish whether these latter lesions may represent completely filled cysts or whether they have arisen as a cyst-independent neoplasm. Epithelial cells lining simple and atypical cysts (Fig 4N and O) and neoplastic cells (Fig 4P) displayed frequent labelling for the proliferation marker Ki67. Simple cysts, atypical cysts and neoplasms all displayed high nuclear immunoreactivity for HIF1α and HIF2α (Fig 4Q–T) verifying that these lesions are derived from *Vhl* null cells. While it is not possible to assay for loss of p53 protein by immunohistochemistry due to the fact that p53 is not detectable in normal kidney cells, PCR genotyping of laser capture micro-dissected simple cysts, atypical cysts and neoplasms demonstrated that the recombined *Trp53* and *Vhl* alleles were present in cells in these lesions (Supporting Information Fig 3). The non-recombined *Trp53* floxed and *Vhl* floxed alleles were also detected, likely due to presence of wild-type (Vhl^fl/fl^;Trp53^fl/fl^) stromal, inflammatory or vascular cells in these lesions.

Immunohistochemical staining using antibodies against NaPi2 (proximal), NCC (distal), THP (thick ascending loop of Henle) and AQP2 (collecting ducts) to mark different tubule segments revealed that most simple cysts express one of these markers (Supporting Information Fig 4), demonstrating that cysts arise from different nephron segments. Very rarely, remnants of the glomerulus could be observed in simple cysts (data not shown), suggesting that these cysts had arisen from the tubular epithelium at the urinary pole of the glomerulus. However, atypical cysts and neoplasms were always negative for all of the tubular markers (Supporting Information Fig 4), preventing assessment of the tubular segment of origin of these lesions and suggesting that the transition to tumour formation involves some degree of de-differentiation. Unlike the findings reported for some precursor lesions in human VHL patient kidneys (Esteban et al, 2006), *Vhl* mutant cystic lesions and neoplasms in the mouse retain expression of the epithelial marker E-cadherin and do not display the mesenchymal marker vimentin (Supporting Information Fig 5).

Thus, *Vhl* and *Trp53* double deletion does not automatically cause proliferative dysregulation of kidney epithelial cells *in vivo* but eventually leads to the evolution of lesions that appear to follow a pathway of simple cyst to atypical cyst to neoplasm that is similar to the proposed disease progression model in kidneys of patients with an inherited *VHL* mutation. Given the apparent morphological similarities and overlapping spectrum of development of atypical cysts and neoplasms, these lesions were grouped together and considered as being distinct from simple cysts in the analyses in the remainder of this study.

Vhl^Δ/Δ^Trp53^Δ/Δ^ mice also displayed a variety of dysplasias and tumours in the genital–urinary tract. Deletion of *Trp53* alone caused a moderate disorganisation of the epithelia in epididymal tubules, predominantly in tubules of the corpus and cauda of the epididymis, with an age-dependent accumulation of aberrant nuclei and multi-nucleated cells (Wild et al, 2012, Supporting Information Fig 6E). Epididymides from Vhl^Δ/Δ^Trp53^Δ/Δ^ mice appeared externally normal in the first months of life (Supporting Information Fig 6B) but histological analysis of aged cohorts revealed that they displayed a qualitatively more severe phenotype of nuclear abnormalities than the Trp53^Δ/Δ^ mice (Supporting Information Fig 6F). At 11–13 months of age, the epididymides of all male Vhl^Δ/Δ^Trp53^Δ/Δ^ mice, but not of control or single mutant mice, displayed benign growths (Supporting Information Fig 6H). These growths were predominantly due to squamous metaplasia (Supporting Information Fig 6I) and extensive epithelial dysplasia (Supporting Information Fig 6J). These lesions are histologically identical to those arising in Vhl^Δ/Δ^Pten^Δ/Δ^ mice (Frew et al, 2008a). Epididymides also frequently displayed fibrosis, inflammation, foreign body reactions and metaplastic stromal changes, likely as a result of the blockage of tubules by dysplasia and squamous metaplasia. One mouse developed an epididymal clear cell papillary cystadenoma (Supporting Information Fig 7A) that appeared histologically identical to the cystadenomas that arise at high frequency in patients with an inherited *VHL* mutation. Vesicular glands of Vhl^Δ/Δ^Trp53^Δ/Δ^ mice (Supporting Information Fig 6L), but not of Vhl^Δ/Δ^ or Trp53^Δ/Δ^ mice (not shown), were malformed. In contrast to the normal single layered epithelium, vesicular glands of Vhl^Δ/Δ^Trp53^Δ/Δ^ mice displayed a disorganized epithelium characterized by multiple convoluted layers of epithelial cells and the formation of gland-like structures (Supporting Information Fig 6P). This phenotype increased in severity with age and two mice exhibited carcinomas in the vesicular gland (Supporting Information Fig 7C). The uterus in all genotypes developed normally (Supporting Information Fig 6R) and displayed a normal organisation of lumenal and glandular endometrial epithelium (Supporting Information Fig 6V). In older Vhl^Δ/Δ^Trp53^Δ/Δ^ mice, small foci of disorganized and multilayered epithelial cells could frequently be observed. Consistent with this, one mouse developed a high-grade carcinoma of the endometrium (Supporting Information Fig 7E) and another a high-grade squamous carcinoma of the upper cervix (Supporting Information Fig 7G). One mouse displayed a high-grade carcinoma that most likely arose in the urothelium of the renal pelvis and which had also metastasized to the lungs and liver (Supporting Information Fig 7I).

Collectively, these findings demonstrate that mutation of the *Vhl* and *Trp53* tumour suppressor genes ultimately causes dysregulation of epithelial cell proliferation and the evolution of dysplastic and malignant lesions in multiple tissues in mice.

### Cooperating pathways in tumour formation in *Vhl/Trp53* double mutant mice

**Figure 5:**
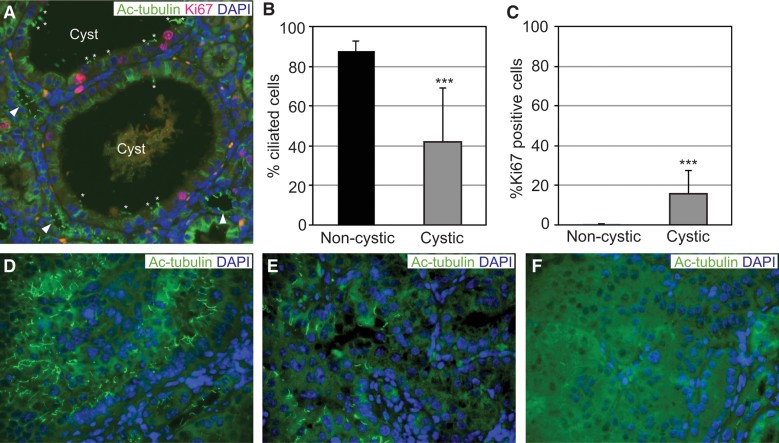
Reduced frequency of primary cilia in cysts **A.** Immunofluorescence staining of formalin-fixed paraffin embedded tissue for acetylated tubulin (green) to mark primary cilia, Ki67 (red) to mark proliferating cells and DAPI (blue) to mark nuclei in a cortical section of kidney from a Vhl^Δ/Δ^Trp53^Δ/Δ^ mouse. Arrowheads point to adjacent normal tubules showing a normal frequency of ciliated cells and * highlight primary cilia in cysts. Note the high frequency of Ki67 negative cells that lack a primary cilium.**B,C.** Quantification of percentage of epithelial cells displaying a primary cilium (B) or staining for Ki67 (C) in non-cystic tubules (*n* = 18) or simple cysts (*n* = 39) in Vhl^Δ/Δ^Trp53^Δ/Δ^ mice. Mean and SD is shown, ***p < 0.001 Student's *t*-test.**D–F.** Examples of neoplasms displaying varying frequencies of primary cilia.

Since we have previously shown a connection between loss of the primary cilium and cyst formation in VHL disease (Frew et al, 2008b; Thoma et al, 2007), we examined whether epithelial cells lining simple cystic lesions that arise in *Vhl/Trp53* double mutant mice displayed a similar loss of primary cilia. Visualising the primary cilium using an antibody against acetylated tubulin revealed that only 40% of cystic epithelial cells but almost 90% of cells in non-cystic tubules displayed a primary cilium (Fig 5A and B). Since only non-proliferating cells exhibit a primary cilium we asked if this reduction in cilia frequency was simply due to the increased proliferation of cystic epithelial cells by staining for Ki67, which labels proliferating cells in all cell cycle stages. On average, 18% of cystic epithelial cells stained positively for Ki67 (Fig 5C), a far lower frequency than the frequency of cells lacking a cilium. Indeed, dual colour immunofluorescence staining experiments revealed that many Ki67 negative cystic cells lacked primary cilia (Fig 5A) demonstrating that the loss of the primary cilium is likely a consequence of loss of pVHL and not an indirect consequence of cellular proliferation and might therefore be causal to cyst formation. Neoplasms displayed a mixed phenotype with respect to cilia, some displayed a very high frequency of ciliated cells (Fig 5D), some displayed an intermediate frequency (Fig 5E) and some were almost completely devoid of cilia (Fig 5F).

**Figure 6:**
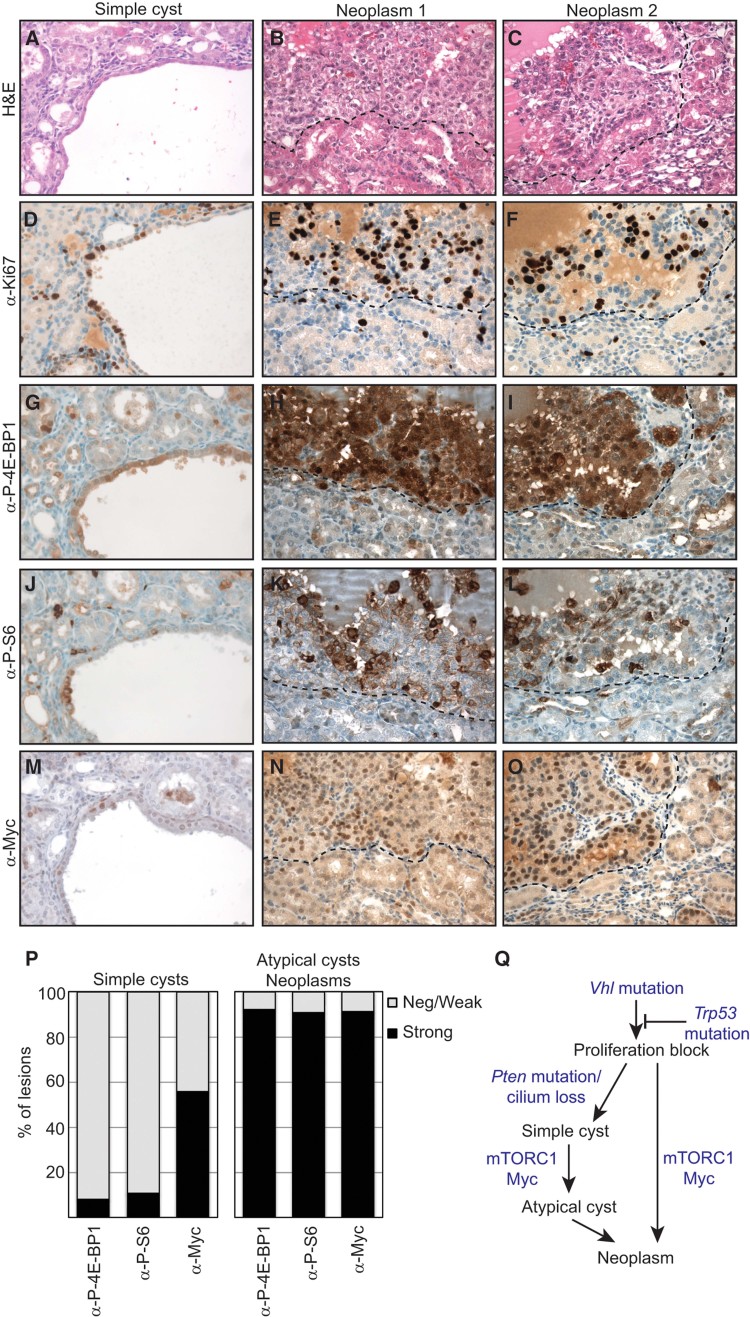
mTORC1 activation and Myc expression signature in atypical cysts and neoplasms **A–O.** Serial sections of a simple cyst (A,D,G,J,M) and two neoplasms (B,E,H,K,N and C,F,I,L,O) stained with H&E (A–C) or stained immunohistochemically for Ki67 (D–F), phospho-Thr37/46-4E-BP1 (G–I), phospho-Ser240/244-ribosomal S6 protein (J–L) or Myc (M–O). Dotted lines indicate the boundary of normal tissue and neoplasms.**P.** Quantification of the percentage of simple cysts (*n* = 68–185) or atypical cysts and neoplasms (*n* = 34–51) that display higher levels (strong) of staining than adjacent normal tissue in the same section (negative/weak).**Q.** Model summarising the proposed sequence of morphological and molecular alterations involved in formation of ccRCC. For details see the Discussion Section.

We have previously demonstrated that one pathway to cilia loss involves both inactivation of pVHL and inhibition of GSK3β (Frew et al, 2008b; Thoma et al, 2007), which can occur via hyperactivation of the PI3K signalling pathway (Frew et al, 2008b). However, immunohistochemical staining using antibodies against phospho-Thr37/46-4E-BP1 (P-4EBP1) (Fig 6G–I) and phospho-Ser240/244-ribosomal S6 protein (P-S6) (Fig 6J–L), two sensitive and robust downstream markers of activation of the PI3K-mTORC1 signalling pathway, revealed that only about 6–8% of simple cysts displayed mTORC1 pathway activation above levels seen in histologically normal tubules in the same mice (Fig 6P). Interestingly, atypical cysts and neoplasms were almost always strongly positive for both of these markers (Fig 6P). Approximately half of all simple cystic lesions and almost all atypical cysts or neoplasms displayed elevated levels of the pro-proliferative Myc protein (Fig 6M–P). Thus, multilayered or papillary growth of *Vhl/Trp53* mutant cells into the lumen of cysts or growth as a solid neoplasm correlates with the acquisition of the pro-proliferative signature of mTORC1 activation and Myc expression.

## DISCUSSION

We show that *TP53* is mutated in a subset of sporadic human ccRCCs and demonstrate genetically that *Trp53* mutation allows *Vhl* null MEFs to escape senescence and proliferate in an immortalized manner. We also show that combined deletion of *Vhl* and *Trp53* in mice results in the formation of simple and atypical cysts, as well as neoplastic lesions in kidneys and causes tumours to form in other genital tract tissues.

The long latency of tumour formation observed in mice (1 year) is consistent with our primary cell culture data showing that *Vhl/Trp53* mutation causes immortalization but not transformation of MEFs. These findings clarify previous contradictory reports concerning the role of p53 in regulating senescence following loss of *Vhl* in mouse fibroblasts (Welford et al, 2010; Young et al, 2008). *Vhl* null cells nonetheless exhibit a lower proliferation rate than *Vhl* wild-type cells, even in the background of loss of the p53 and pRB cell cycle checkpoints, implying that there may be additional cellular responses that represent barriers that prevent full transformation of *Vhl/Trp53* mutant cells. We suggest that the increase in aneuploidy observed in *Vhl/Trp53* null MEFs might potentially represent a mechanism that could contribute to cellular transformation and tumour evolution *in vivo*.

Our findings strengthen the model derived from studies of human VHL patients that ccRCCs can form via cyst-dependent and cyst-independent pathways (Fig 6Q). *Vhl/Trp53* mutant mice develop an apparent spectrum of cystic lesions beginning with simple cysts lined by a single layer of epithelial cells, followed by atypical cysts that display micro-papillary epithelial growths that project into the lumen of the cyst and finally cysts that are almost entirely filled with neoplastic growth. About half of the neoplasms are a solid mass of cells, preventing assessment of whether they arise via a cystic precursor lesion or not. *Vhl/Trp53* neoplastic lesions display several features of human ccRCC including clear cell-like changes, HIFα stabilisation and high rate of proliferation, but differ in that they exhibit a low nuclear grade and do not invade surrounding tissue. The lack of a capsule surrounding the neoplasms and absence of extra-renal metastases speaks against a malignant ccRCC lesion. *Vhl/Trp53* mutant neoplasms also frequently grow in a micro-papillary pattern, akin to papillary renal cell carcinomas. While the precursor lesions of human renal carcinomas are poorly characterized in general, in papillary type I and type II tumours the size of the lesion is the sole definitive distinguishing criteria. Lesions smaller than 5 mm are classified as adenomas and larger lesions are carcinomas (Eble et al, 2004). Taking the relative sizes of the human and mouse kidney into account, many of the neoplasms in our model would be classified as carcinomas under this definition. Because of the mixed features of the *Vhl/Trp53* null neoplasms we classify these tumours simply as renal neoplasms, rather than as a specific sub-type of renal cell carcinoma.

Epithelial cells lining simple cystic lesions display a reduced frequency of primary cilia, similar to cysts in human VHL patients (Thoma et al, 2007), further supporting the involvement of pVHL in maintenance of primary cilia and suppression of cyst formation. However, in contrast to *Vhl/Pten* mutant mice (Frew et al, 2008b), in *Vhl/Trp53* mutant mice, these simple cysts do not display evidence of over-activation of the PI3K signalling pathway or inactivating phosphorylation of GSK3β (unpublished observations), implying that there may be other unidentified pathways that cooperate with pVHL in maintenance of the primary cilium. In contrast to simple cysts, atypical cysts and neoplasms display hyperactivation of mTORC1 signalling. Since both lesions are characterized by disorganized patterns of cellular growth it is noteworthy that mTORC1 activation has been shown to induce a translational program that promotes cellular invasion (Hsieh et al, 2012). Hyperactivation of mTORC1 predicts poor outcome in ccRCC patients and mTORC1 inhibitors show clinical efficacy against ccRCC (Hudes, 2009). Atypical cysts and neoplasms almost invariably also display high levels of Myc protein. Upregulation of *MYC* expression is common in ccRCC and amplification of *MYC* predicts poor outcome in human ccRCC patients (Monzon et al, 2011; Tang et al, 2009). The combination of *Vhl/Trp53* double mutation with a pro-proliferative signature of mTORC1 activation and high Myc expression therefore correlates with the transition to a neoplastic state.

While approximately 1 in 10 ccRCC tumours harbour *TP53* mutations, in many epithelial malignancies the *TP53* mutation frequency is much higher (50–90%). In ccRCC, several mechanisms have been proposed to act to compromise p53 function, potentially alleviating the selective pressure for *TP53* mutation or deletion during tumour formation. USP10 normally de-ubiquitinates p53 in response to DNA damage, opposing the action of Mdm2 and allowing p53 protein accumulation (Yuan et al, 2010). Interestingly, 90% of ccRCC express lower than normal levels of USP10, possibly leading to reduced p53 activation (Yuan et al, 2010). pVHL itself has been implicated as a factor important for full p53 activation by promoting the recruitment of the p300 acetylase and ATM kinase to p53 (Roe et al, 2006). Knockdown of *VHL* expression reduced p53 activity in response to DNA damage and reintroduction of pVHL expression in *VHL*-deficient RCC cells enhanced damage-induced activation of p53 (Roe et al, 2006). Downstream of loss of pVHL function, activation of HIFα transcription factors may also act to compromise p53 activity. The hypoxia-inducible *PAX2* gene is a transcriptional repressor of *TP53* and is highly upregulated in *VHL* mutant cells and ccRCCs (Luu et al, 2009; Stuart et al, 1995). Elevated HIF2α levels in *VHL*-mutant ccRCC are proposed to induce growth factor expression leading firstly to the AKT-mediated phosphorylation of HDM2, promoting its ability to degrade p53 (Roberts et al, 2009) or secondly to the suppression of formation of reactive oxygen species which reduce p53 activation (Bertout et al, 2009). *PBRM1* is mutated in 41% of ccRCCs (Varela et al, 2011) and has been shown to be necessary for induction of senescence by p53 (Burrows et al, 2010), thus potentially abrogating part of p53's tumour suppressing activity in the kidney. In our hands however, knockdown of *Pbrm1* failed to alleviate proliferative arrest following *Vhl* knockout in MEFs (unpublished observations). Similarly, *SETD2* is mutated in a small fraction of ccRCCs (Dalgliesh et al, 2010) and has been suggested to regulate a subset of p53 target genes (Xie et al, 2008). Thus, p53 function may either be lost by mutation or compromised by other mechanisms in a large proportion of *VHL*-negative ccRCCs.

It will be important to clarify when *TP53* mutations arise during the process of tumour initiation and progression. In this regard, a study of four ccRCCs utilized deep sequencing of the tumour DNA population to reconstruct the molecular evolutionary history of the tumours (Gerstung et al, 2012). In one of these tumours a single *TP53* truncation mutation was present at about one-fifteenth the frequency of a single *VHL* frameshift mutation, implying that the *TP53* mutation was an event that occurred secondarily to an initiating *VHL* mutation and that it resulted in the formation of a *VHL/TP53* double mutant sub-clone of the tumour cell population. This finding supports the notion that genetic cooperation between *VHL* and *TP53* mutations promotes tumour progression. Similar analyses of larger numbers of ccRCC samples from different stages of disease progression would test how representative this initial finding is for ccRCCs in general.

In summary, we present strong evidence to support the idea that loss of function of *VHL* and *TP53* is a *bone fide* tumour promoting combination and describe a mouse model that recapitulates many of the steps involved in the formation of *VHL* mutant kidney tumours in humans.

## MATERIALS AND METHODS

### Mouse genetics

Previously described *Ksp1.3-Cre/+*; *Vhl*^*fl/fl*^ (Frew et al, 2008b) and *Ksp1.3-Cre/+*; *Trp53*^*fl/fl*^ (Wild et al, 2012) mouse strains were interbred to generate *Ksp1.3-Cre/+*; *Vhl*^*fl/fl*^; *Trp53*^*fl/fl*^ mice. Non-Cre transgenic littermate mice served as controls for all cohorts. Wild-type cells were isolated from C57BL/6 embryos.

### Analyses of human ccRCCs

Tissue samples were from the University Hospital of Zurich (Zurich, Switzerland). The study was approved by the local ethics commission (reference number StV 38-2005). Haematoxylin and eosin stained sections of all paraffin embedded ccRCC specimens were reviewed by H.M. DNA extraction and *VHL* sequencing were performed as previously described (von Teichman et al, 2011). The primers used for PCR and sequencing of *TP53* exons 5–8 are listed in Supporting Information Table S1. PCR was performed with 40 cycles consisting of denaturation at 94°C for 45 s, annealing at 58°C for 45 s and extension at 72°C for 45 s. *VHL* and *TP53* mutations were validated by an independent PCR and sequence analysis. Paraffin sections (2.5 µm) were treated using Ventana Benchmark XT (Tuscon, AZ, USA) or BOND-MAX (Leica Microsystems, Wetzlar, Germany) automated systems. Immunostainings for CAIX, GLUT1 and HIF1α were performed as recently described (Dahinden et al, 2010; Luu et al, 2009). Nuclear HIF1α and membranous CAIX, GLUT1 expression were defined positive if at least 5% of tumour cells showed weak (+1) or strong (+2) staining.

### Assays of MEFs

MEFs were isolated from relevant floxed strains and aliquots were frozen at passage 2. *Trp53*^*−/−*^ MEFs were a kind gift from Scott Lowe. Cells were cultured either in conventional cell culture incubators at atmospheric oxygen or at 5% oxygen or were cultured in a darkened oxygen glove-box incubator (INVIVO_2_ 400, Ruskinn) at 5% oxygen in which medium and PBS were equilibrated for 2 h prior to splitting of cells to ensure that cells were exposed to constant oxygen tension throughout the experiment. For proliferation assays, cells were seeded at densities of either 2 × 10^5^ or 3 × 10^5^ cells per 6 cm dish in triplicate dishes and counted after 3 days before reseeding at the same density for the next passage. All proliferation assays shown in the Figures are representative of at least three independent experiments. Wild-type and *Vhl*^*fl/fl*^ MEFs were transformed by transfection with a plasmid expressing SV40 large T-Antigen (Addgene, pBSSVD2005) and pools of cells that formed colonies after plating at low density were harvested to generate cell lines. Cells were infected with adenoviruses expressing GFP (Vector Biolabs, 1060) or Cre-GFP (Vector Biolabs, 1700), retroviruses (LMP) expressing non-silencing hairpin or miR30-shRNA against *Trp53* (Dickins et al, 2005), lentiviruses (LKO.1) expressing non-silencing hairpin (Addgene, 10879) or shRNA against *Vhl* (Open Biosystems, TRC0000009735) (Thoma et al, 2007). For lentiviral-mediated knockdown of *Trp53*, we generated a vector (pLenti X1 Puro DEST, Addgene 17297) containing the U6 promoter (derived from pENTR/pSM2 (U6), Addgene 17387) driving expression of a previously described (Dickins et al, 2005) miR30 format shRNA against *Trp53* (1224) or expressing an empty (ns) miR30 backbone. Infections were followed after 48 h by puromycin selection (4 µg/ml) where appropriate. Genotyping for the floxed or recombined *Vhl* and *Trp53* alleles were performed as described (Biju et al, 2004; Jonkers et al, 2001). Flow cytometry (Frew et al, 2002) and counting of aberrant anaphases (Burds et al, 2005) were performed as described.

**The paper explained**

PROBLEM:

The cooperating genetic events that lead to the formation of clear cell renal cell carcinoma (ccRCC), the most frequent form of kidney cancer in humans, remain unclear. While the vast majority of familial and sporadic forms of ccRCC harbour biallelic inactivation of the von Hippel–Lindau tumour suppressor gene (*VHL*), loss of *VHL* function alone in humans and in mice is insufficient to cause kidney tumour formation. It is presumed that other genetic events must cooperate with loss of *VHL* to cause ccRCC but these cooperating mutations remain poorly understood.

RESULTS:

Here we identify loss of function mutations in *TP53* in a subset of sporadic human ccRCCs and show that kidney-specific combined deletion of *Vhl* and *Trp53* leads to the formation of cysts and tumours in mice, recapitulating the precursor lesions and cellular and molecular alterations that are involved in the formation of *VHL* mutant ccRCC in humans.

IMPACT:

These findings provide the first demonstration that secondary genetic alterations can cooperate with loss of *VHL* to cause kidney tumour formation and implicate *TP53* mutations in the pathogenesis of a subset of human ccRCC.

### Real-time PCR

Real-time PCR was performed as described (Frew et al, 2008b) using the following primer pairs: *18S rRNA* (5′-TGGCCGACCATAAACGATGCC-3′, 5′-TGGTGGTGCCCTTCCGTCAAT-3′), *Mad2* (5′-GTGGCCGAGTTTTTCTCATTTG-3′, 5′-AGGTGAGTCCATATTTCTGCACT-3′).

### Kidney epithelial cell proliferation assays

Kidneys were dissected from 2-month-old floxed mice. After removing the capsule under sterile conditions, kidneys were mashed with a razor blade on ice and digested in collagenase II (Gibco) and soya trypsin inhibitor (Gibco) solution at 37°C for 30 min. The cell suspension was filtered through a 70 µm cell strainer and washed in HBSS + 5% FCS. Erythrocytes were lysed for 1 min using standard ACK buffer. Cells were resuspended in complete K-1 culture medium [Dulbecco's modified Eagle's medium (DMEM):Hams F12] (50:50), supplemented with 0.5% foetal calf serum, hormone mix [5 µg/ml insulin, 1.25 ng/ml prostaglandin E_1_ (PGE_1_), 34 pg/ml triiodothyronine, 5 µg/ml Apo-transferrin, 1.73 ng/ml sodium selenite and 18 ng/ml of hydrocortisone] and 25 ng/ml epidermal growth factor (EGF). Cells were counted and seeded at a density of 1 × 10^6^ cells on standard 100 mm plastic tissue culture plates. After 5–6 days in culture, cells were infected with adenoviruses expressing GFP (Vector Biolabs, 1060) or Cre-GFP (Vector Biolabs, 1700). Sulforhodamine B (SRB) proliferation assay was performed in 96-well format as described (Vichai & Kirtikara, 2006). Briefly, primary kidney epithelial cells were cultured in K-1 medium containing 10% foetal calf serum for 2 days before seeding for the SRB assay. 2 × 10^3^ cells per well were seeded and fixed in 5% w/v trichloracetic acid at the indicated time points. Cells were stained in 0.057% w/v SRB solution and air dried. SRB was solubilized by incubation in 10 mM Tris base solution (pH 10.5) and OD was measured at 540 nm in a micro-plate reader.

### Antibodies, Western blotting, immunofluorescence and immunohistochemistry

Western blotting, immunohistochemistry or immunofluorescence were conducted using previously described methods (Frew et al, 2008b) and the antibodies against the following epitopes: Acetylated tubulin (Sigma, #T6793), Actin (Sigma-Aldrich, A2228), AQP2 (Wagner et al, 2008), Aurora A (Abcam, ab13824), BubR1 (BD Biosciences, 612502), CDK-2 (Santa Cruz, sc-163-g), Cenp-E (Meraldi et al, 2004), E-cadherin (Abcam, ab11512), phospho-Thr37/46-4E-BP1 (Cell Signaling Technology, #2855), HIF1α (Novus Biologicals, NB100-105), HIF2α (Pollard et al, 2007, PM8), Ki67 (DakoCytomation, TEC-3), Mad2 (Bethyl Laboratories, A300301A), Myc (Epitomics, Y69), p53 (Novocastra, NCL-p53-CM5p), NaPi2 (Custer et al, 1994), NCC (Millipore, AB3553), phospho-Ser240/244-ribosomal S6 protein (Cell Signaling Technology, #2215), THP (Santa Cruz Biotechnology, sc-20631), pVHL(m)_CT_ antibody (Hergovich et al, 2003), pVHL (Santa Cruz, sc-5575), Vimentin (Cell Signaling Technology, #5741).

## For more information

For information about the VHL Family Alliance for patients with inherited VHL disease:

http://www.vhl.org

For general information about kidney cancer subtypes and treatments:

http://www.cancer.gov/cancertopics/types/kidney

For information about the genetic mutations that have been found in ccRCC:

http://www.sanger.ac.uk/perl/genetics/CGP/cgp_viewer?action=study;study_id=321

http://www.sanger.ac.uk/genetics/CGP/Studies/Renal/

http://www.sanger.ac.uk/perl/genetics/CGP/cosmic?action=byhist&s=4&hn=carcinoma&sn=kidney&sh=clear_cell_renal_cell_carcinoma

## Supplementary material

As a service to our authors and readers, this journal provides supporting information supplied by the authors. Such materials are peer reviewed and may be re-organized for online delivery, but are not copy-edited or typeset. Technical support issues arising from supporting information (other than missing files) should be addressed to the authors.
