# Supplementary material for: Combined mutation of Vhl and Trp53 causes renal cysts and tumours in mice
Source: EMBO Mol Med. 2013 Apr 22;5(6):949–64. doi: 10.1002/emmm.201202231 (PMC3779454; doi:10.1002/emmm.201202231)
Supplement: Supplementary file 1 [file emmm0005-0949-SD1.pdf]

Combined mutation of Vhl and Trp53 causes renal cysts and tumours in mice

Joachim Albers, Michal Rajski, Désirée Schönenberger, Sabine Harlander, Peter Schraml, Adriana von Teichman, Strahil Georgiev, Peter J. Wild, Holger Moch, Wilhelm Krek and Ian J. Frew

Corresponding author: Ian Frew, University of Zurich

Review timeline:

Submission date:	02 November 2012
Editorial Decision:	09 January 2013
Revision received:	08 March 2013
Accepted:	12 March 2013

Transaction Report:

(Note: With the exception of the correction of typographical or spelling errors that could be a source of ambiguity, letters and reports are not edited. The original formatting of letters and referee reports may not be reflected in this compilation.)

Editor: Roberto Buccione

1st Editorial Decision 09 January 2013

Thank you for the submission of your manuscript to EMBO Molecular Medicine.

In this case we experienced unusual difficulties in securing three appropriate reviewers in a timely manner. Since we have received consistent evaluations from 2 Reviewers, and cannot justify a further delay, I have decided to proceed based on these evaluations.

You will see that both Reviewers are generally supportive of your work and underline its considerable potential interest. Reviewer 1, however, does express a few concerns that require your attention and action. For this reason, publication of the paper cannot be considered at this stage.

Specifically, Reviewer 1 would like to see data on p53 status in the Vhl knock down experiments illustrated in Figure 1. Furthermore, s/he feels that a more appropriate approach would have been to use MEFs from a Vhlfl//lTrp53-/- embryo vs. Trpfl/fl and/or Trp-/fl. I would encourage you to develop the study as far as realistically possible in this sense to strengthen your findings. Reviewer 1 also notes that the criteria for cyst labelling are not clear. Finally, s/he requires a number of other clarifications and improvements that also need your action.

We would thus be pleased to consider a suitably revised version.

Please note that it is EMBO Molecular Medicine policy to allow a single round of revision only and

that, therefore, acceptance or rejection of the manuscript will depend on the completeness of your responses included in the next, final version of the manuscript.

I look forward to receiving your revised manuscript as soon as possible.

***** Reviewer's comments *****

Referee #1 (Comments on Novelty/Model System):

This is an elegant, carefully executed and detailed piece of novel research. It is clear and well written. Even though the authors might have tried to extend some of the analyses into human cells/ tumours beyond what they did, the body of work stands well on its own as a mouse model and to highlight the complexity of renal neoplasia (ie that there are multiple events that are not mutually exclusive that can lead to renal cancer) and that there is a real case for considering Trp53 as a secondary event in renal cancer following inactivation of Vhl.

Referee #1 (General Remarks):

This is an elegant, carefully executed and detailed piece of research. It is clear and well written. There are a few minor formatting & other errors; but these will be dealt with by editorial procedures (eg figure 5 legend).
My only general comment is that the authors might have tried to extend some of the analyses into human cells/ tumours beyond what they did; but the body of work stands well on its own as a mouse model and to highlight the complexity of renal neoplasia (ie that there are multiple events that are not mutually exclusive that can lead to renal cancer) and that there is a real case for considering Trp53 as a secondary event in renal cancer following inactivation of Vhl .

Minor comments:

(1)In experiments in figure 1 the authors should include the p53 status of the cells for completeness; not just Vhl. This could be done in the text. Two reasons for asking this-1) sometimes the Adcre does not delete efficiently all floxed alleles at the same time and, 2) it would have been interesting to see if any of the cells were hetfloxed for p53 while Vhl was deleted and whether this conferred any different proliferation or other status on the cells.

(2) It may not have been feasible for the authors to do this; but I would have been inclined to use MEFs from a Vhlfl/flTrp53-/- embryo versus eg littermates with Trpfl/fl &/or Trp-/fl.

(3) There is a huge attention to detail in the work; but in some cases this is a little excessive eg I do not think that all the lanes needed to be shown for Fig 1M.
The authors have used actin and cdk2 as loading controls interchangeably throughout the paper (Figures 1-3) without explanation that these are loading controls, nor why one rather the other, is used. Please explain.

(4) Also, the reason for measuring proliferation rate in different ways is not clear- doubling times versus SRB assay (Figures 1 &2). Again, please explain.

(5) Figure 4 and text. It would be helpful if the authors could define the criteria for labelling of the cysts eg comparing glomerular versus tubular. One obvious difference between the cysts shown in

4G & 4H is the contour of the cyst. The status of the epithelium lining these cysts is difficult to discern at the magnifications shown. Also to define what they mean by 'rare' with respect to the glomerular cysts. It might be useful to have a low power image of multiple cysts in this figure or a supplementary figure.

(6) Discussion of the cystic lesions observed in the double KO mice might be better described in a table (pg 9-10). Also and importantly the authors should show evidence that Vhl and p53 have been effectively deleted by the Kspcre. Some heterogeneity in the effectiveness of deletion of both Vhl and p53 may account in part for the heterogeneity of cystic lesion.

(7) Figure 5- analysis of cilia with acetylated tubulin and by Immunofluorescence. Please could the authors specify how this was done- I assume on frozen sections; but it would be helpful to know.

(8) One issue that remains unresolved in linking loss of both Vhl and p53 in renal carcinoma is the time course of loss of p53; whether that is a bi-phasic event and when in the course of development of the carcinoma loss occurs. Perhaps the authors might have suggestions to make about this.

Referee #2 (Comments on Novelty/Model System):

The authors test the combined VHL + p53 inactivation in an animal model. The choices of combination are reasonable and clinically relevant. The techniques are standard.

Referee #2 (General Remarks):

This is a very nice piece of work testing in an animal model the clinically relevant combination of p53 and Vhl inactivation in the kidney. Technically the work is outstadning. The data suggest that despite the combined inactivation tumorigencity is low. The observation is discussed by the authors.

1st Revision - authors' response 08 March 2013

Referee # 1

Minor comments:

(1) In experiments in figure 1 the authors should include the p53 status of the cells for completeness; not just Vhl. This could be done in the text. Two reasons for asking this-1) sometimes the Ad-Cre does not delete efficiently all floxed alleles at the same time and, 2) it would have been interesting to see if any of the cells were het floxed for p53 while Vhl was deleted and whether this conferred any different proliferation or other status on the cells.

We agree and have added p53 western blots for Figures 1H, J, L and N. We also analysed recombination of the *Trp53* locus in the clonal cell line studies in Figure 1M using PCR and found that all 135 cell lines have homozygously recombined the floxed *Trp53* allele. All of these analyses show that p53 expression is completely lost in bulk populations and that *Trp53* is homozygously recombined in all of the clones upon Ad-Cre treatment. We have conducted dozens of experiments (see eg Fig 2A and Fig 3A) that all show that both pVHL and p53 expression are equally lost upon Cre treatment.

(2) It may not have been feasible for the authors to do this; but I would have been inclined to use MEFs from a Vhlfl/flTrp53-/- embryo versus eg littermates with Trpfl/fl &/or Trp-/fl.

We agree that it is important to only compare proliferation rates between littermate embryos since independent preparations of MEFs even from different litters of wild type mice can display large variations in proliferation rates. For this reason we always present separate growth curves in which we compare the relative (rather than absolute) effects of GFP and Cre treatment within a particular floxed background. All of our MEFs are derived from pools of embryos from a single litter, removing some of the potential prep-to-prep variation in proliferation rates. While we do not have a null *Trp53* allele, we have previously tried several times to obtain litters from intercrosses of $Vhl^{fl/+}Trp53^{fl/fl}$ mice in the hope of obtaining littermate embryos that were $Vhl^{+/+}Trp53^{fl/fl}$ and $Vhl^{fl/fl}Trp53^{fl/fl}$ to perform a similar experiment to that suggested by the reviewer. However, the fact that each of these genotypes occurs at a frequency of 1:4 from this cross and the fact that the Vhl-floxed genetic background generally leads to small litter sizes, prevented us from obtaining the desired embryos. For this reason we conducted the alternative set of experiments described in Figure 1I and Figure 1K that achieve a similar purpose to the experiment proposed by the reviewer, namely allowing clean genetic analysis of the effect of deletion of *Vhl* in an isogenic setting of loss of p53 function (by knockdown in I) and loss of p53/pRB function (by SV40-Tag in K). These experiments gave the same results as the experiments using the floxed alleles, allowing use to conclude as we have in the manuscript.

(3) There is a huge attention to detail in the work; but in some cases this is a little excessive eg I do not think that all the lanes needed to be shown for Fig 1M. The authors have used actin and cdk2 as loading controls interchangeably throughout the paper (Figures 1-3) without explanation that these are loading controls, nor why one rather the other, is used. Please explain.

Based on this suggestion we have altered Fig 1M to show only a selection of clones that are representative of all of the data.

Both CDK2 and actin are very well established loading and transfer controls for western blotting that have been described in countless papers. The reason for using them interchangeably in the paper is not a biological one but simply that the various experiments were performed over a long time period and the availability/quality of the antibodies changed during this time. We have now added an additional

statement in the figure legend for Fig 1F, the first time that the actin antibody is shown in the paper, that "Immunoblotting using an antibody against actin served as a loading and transfer control." A similar statement for Cdk2 was already present for Figure 1B,D, which is the first time that the Cdk2 antibody was shown. For reasons of space in the figure legends we opted to state this only the first time the antibodies are used and not in each subsequent instance.

(4) Also, the reason for measuring proliferation rate in different ways is not clear- doubling times versus SRB assay (Figures 1 &2). Again, please explain.
The reason is that MEFs can be continually passaged and counted at each passage, whereas it is very difficult to obtain large numbers of primary kidney cells and these cannot be harvested and replated as they rapidly undergo de-differentiation upon passaging (but maintain their epithelial character when plated and are allowed to proliferate in the short term on the same dish). The SRB assay provides a very sensitive assay to indirectly quantitate cell numbers in wells, allowing short-term proliferation assays of epithelial cells directly after they have been plated.

(5) Figure 4 and text. It would be helpful if the authors could define the criteria for labelling of the cysts eg comparing glomerular versus tubular. One obvious difference between the cysts shown in 4G & 4H is the contour of the cyst. The status of the epithelium lining these cysts is difficult to discern at the magnifications shown. Also to define what they mean by 'rare' with respect to the glomerular cysts. It might be useful to have a low power image of multiple cysts in this figure or a supplementary figure.
We are thankful to the reviewer for raising these points as we have realized that the key message that we aimed to convey about the cysts was somewhat lost in the initial version of the manuscript. We have reorganized Figure 4 taking into account several of the suggestions of the reviewer. Figure 4G is now a representative low power image showing a region of the kidney containing several simple cysts, an atypical cyst and a neoplasm. We decided to remove the panel (that we initially included for the sake of completeness) that showed what we had previously termed a "glomerular" cyst. This term that we chose is actually somewhat of a misnomer. In very rare cysts (less than 0.5%) we could observe remnants of the glomerulus. These cysts most likely arise due to deletion of *Vhl/Trp53* in the tubular epithelium at the urinary pole of the glomerulus (where we see strong nuclear staining of Hif1a and Hif2a, shown in Supporting Figure 2, indicative of deletion of *Vhl* in these cells). Thus the cysts are not actually cysts of the glomerulus *per se* but of the renal tubule immediately proximal to the glomerulus. Since these cysts are very rare we decided to remove this image from the figure and instead now describe these cysts containing glomerular remnants in the text along with the description of the marker studies that show the origin of the tubular cysts.

The majority of the simple cysts display a single layered epithelial border comprising cuboidal epithelial cells (which varies in appearance a little depending on the section of the nephron from which the cyst arises as well as the size of the cyst). We have included a zoom of a representative section of cystic epithelium from a representative cyst in Figure 4H, as well as a zoom of the appearance of a representative atypical cyst in Figure 4G. We have included a short description of the morphology of the cyst lining cells in the text.

(6) Discussion of the cystic lesions observed in the double KO mice might be better described in a table (pg 9-10). Also and importantly the authors should show evidence that Vhl and p53 have been effectively deleted by the Kspcre. Some heterogeneity in the effectiveness of deletion of both Vhl and

p53 may account in part for the heterogeneity of cystic lesion.

We initially considered presenting a table of different lesions but quickly realized that this becomes unnecessarily complex and the overview of the different types of lesions is easily lost. For this reason we opted to summarise the types of lesions in the text to emphasise the key features and frequencies of these lesions.

Using Hif1α and Hif2α immunohistochemical staining as a surrogate marker of *Vhl* deletion we had shown in the first version of the manuscript that *Vhl* is deleted in all cysts and neoplasms (our previous knockout studies have shown that only homozygous, but not heterozygous *Vhl* deletion, causes HIFα stabilization). Similar attempts to monitor *Trp53* deletion using two different p53 antibodies were not successful as p53 is not detectable in undamaged kidneys and therefore cannot be used as a readout of genetic deletion. For this reason we performed laser capture microdissection to isolate DNA from simple cysts, atypical cysts and neoplastic lesions. PCR was performed to genotype the *Trp53* and *Vhl* loci for recombination of the floxed alleles. All samples displayed the recombined *Trp53* and *Vhl* alleles but also displayed the floxed *Trp53* and *Vhl* alleles. This result has been added as a new Supporting Information Fig 3. While this assay does not allow us to conclusively exclude that some cells in the lesions may be heterozygous for *Trp53* deletion, given the fact that the Cre transgene is active all the way from embryogenesis through adult life, and given the strong evidence from our cell culture studies that the floxed *Vhl* and *Trp53* alleles are equally efficiently deleted upon addition of Cre, plus the HIFα staining data, we conclude that the most likely interpretation is that the cells in the lesions are null for *Vhl* and *Trp53* and the detected *Vhl* and *Trp53* floxed alleles come from the wild type ($Vhl^{fl/fl}$;$Trp53^{fl/fl}$) stromal, immune and vascular cells that are present in these lesions or that are co-dissected from the cyst border due to the imprecision of the LCM technique.

The section of text below was added to the results section on page 10 to summarise these points:
"While it is not possible to assay for loss of p53 protein by immunohistochemistry due to the fact that p53 is not detectable in normal kidney cells, PCR genotyping of laser capture microdissected simple cysts, atypical cysts and neoplasms demonstrated that the recombined *Trp53* and *Vhl* alleles were present in cells in these lesions (Supporting Information Fig 3). The non-recombined *Trp53* floxed and *Vhl* floxed alleles were also detected, likely due to presence of wild type ($Vhl^{fl/fl}$;$Trp53^{fl/fl}$) stromal, inflammatory or vascular cells in these lesions."

(7) Figure 5- analysis of cilia with acetylated tubulin and by Immunofluorescence. Please could the authors specify how this was done- I assume on frozen sections; but it would be helpful to know.

These analyses were performed by staining paraffin sections using a protocol that we have previously described. We have altered the Methods section to better highlight the reference to this method and have included a statement in the legend for Figure 5 stating that these are formalin fixed, paraffin embedded samples. We also corrected a small formatting error that was pointed out by the reviewer (D/D was corrected to Δ/Δ).

(8) One issue that remains unresolved in linking loss of both Vhl and p53 in renal carcinoma is the time course of loss of p53; whether that is a bi-phasic event and when in the course of development of the carcinoma loss occurs. Perhaps the authors might have suggestions to make about this.

We are grateful to the reviewer for raising this question and agree that it is a relevant discussion point for our paper that we had neglected. We have included a new paragraph in the discussion (page 16) that

deals with this point:
“It will be important to clarify when *TP53* mutations arise during the process of tumour initiation and progression. In this regard, a study of four ccRCCs utilized deep sequencing of the tumour DNA population to reconstruct the molecular evolutionary history of the tumours (Gerstung et al, 2012). In one of these tumours a single *TP53* truncation mutation was present at about one fifteenth the frequency of a single *VHL* frameshift mutation, implying that the *TP53* mutation was an event that occurred secondarily to an initiating *VHL* mutation and that it resulted in the formation of a *VHL/TP53* double mutant sub-clone of the tumour cell population. This finding supports the notion that genetic cooperation between *VHL* and *TP53* mutations promotes tumour progression. Similar analyses of larger numbers of ccRCC samples from different stages of disease progression would test how representative this initial finding is for ccRCCs in general.”

Referee #2 (Remarks):

This is a very nice piece of work testing in an animal model the clinically relevant combination of p53 and Vhl inactivation in the kidney. Technically the work is outstanding. The data suggest that despite the combined inactivation tumorigencity is low. The observation is discussed by the authors.

We are very grateful to the reviewer for this strong endorsement of our study.

Accepted 12 March 2013

Please find enclosed the final report on your manuscript. We are pleased to inform you that your manuscript is accepted for publication and is being sent to our publisher to be included in the next available issue of EMBO Molecular Medicine.

***** Reviewer's comments *****

Referee #1 (Comments on Novelty/Model System):

This work is very novel and of excellent technical quality. The revised manuscript has improved this even further and in my opinion there are no ethical issues that may arise from the model organsim (mouse).

Referee #1 (General Remarks):

The original version of the manuscript was of already a high standard though required some small changes. The authors have revised the manuscript (based on the minor changes advised) and further improved its content and suitability for a general audience. I would be very supportive of this work being published without any further revisions (aside from that at the editorial level).
