# Supplementary material for: Combined mutation of Vhl and Trp53 causes renal cysts and tumours in mice
Source: EMBO Mol Med. 2013 Apr 22;5(6):949–64. doi: 10.1002/emmm.201202231 (PMC3779454; doi:10.1002/emmm.201202231)
Supplement: Supplementary file 2 [file emmm0005-0949-SD2.pdf]

Combined mutation of *Vhl* and *Trp53* causes renal cysts and tumours in mice

Joachim Albers, Michal Rajski, Désirée Schönenberger, Sabine Harlander, Peter Schraml, Adriana von Teichman, Strahil Georgiev, Peter J. Wild, Holger Moch, Wilhelm Krek and Ian J. Frew

Supporting Information Table of Contents

Supporting Information Figures 1-7 Pages 1-7

Supporting Information Table 1 Page 8

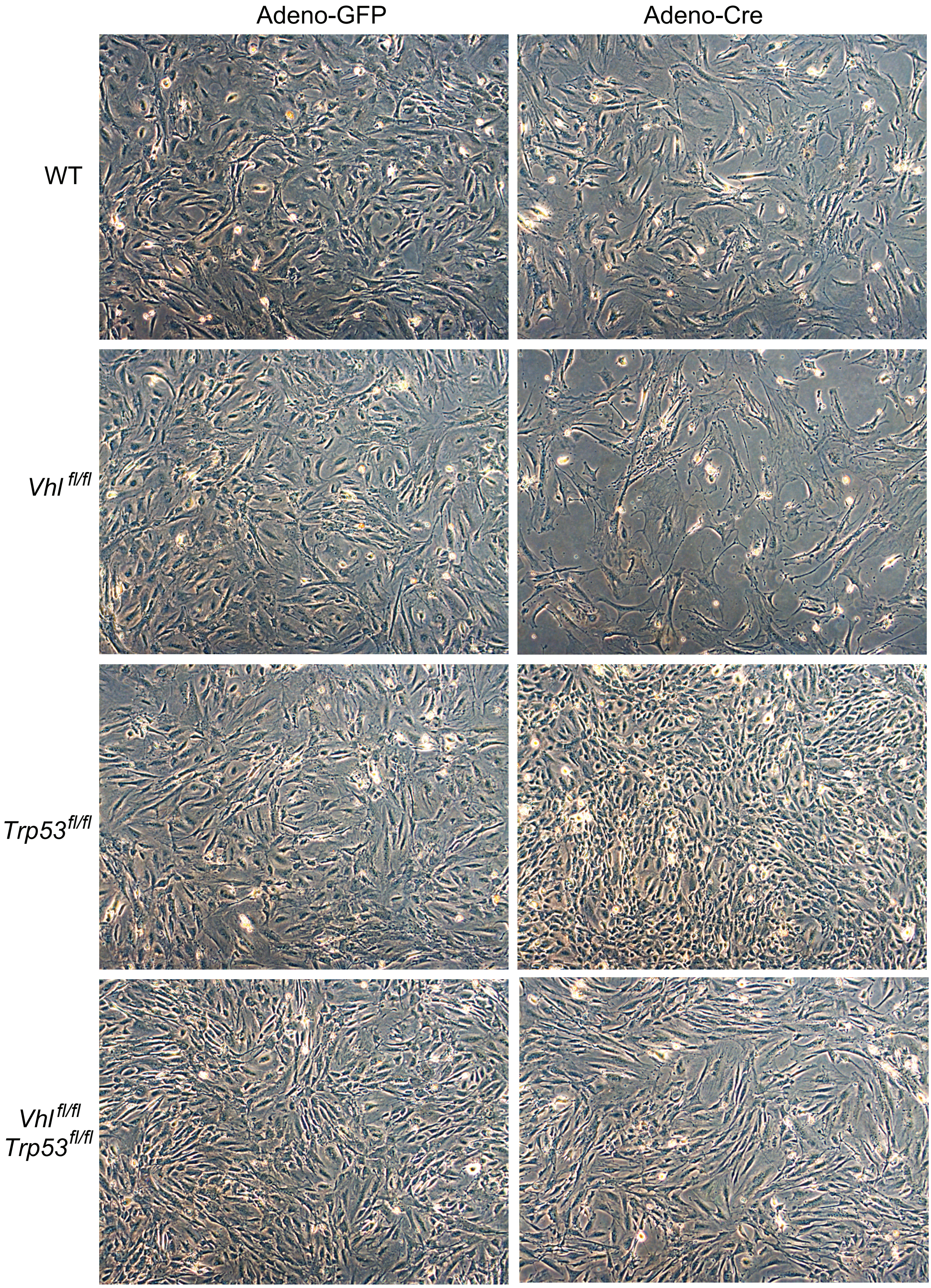

Supporting Information Fig 1. *Trp53* deletion rescues the senescent appearance of *Vhl* null MEFs.

Phase contrast images of wild type, *Vhl*$^{fl/fl}$, *Trp53*$^{fl/fl}$ or *Vhl*$^{fl/fl}$ *Trp53*$^{fl/fl}$ MEFs 10 days after infection with adenoviruses expressing GFP (Adeno-GFP) or Cre-GFP (Adeno-Cre).

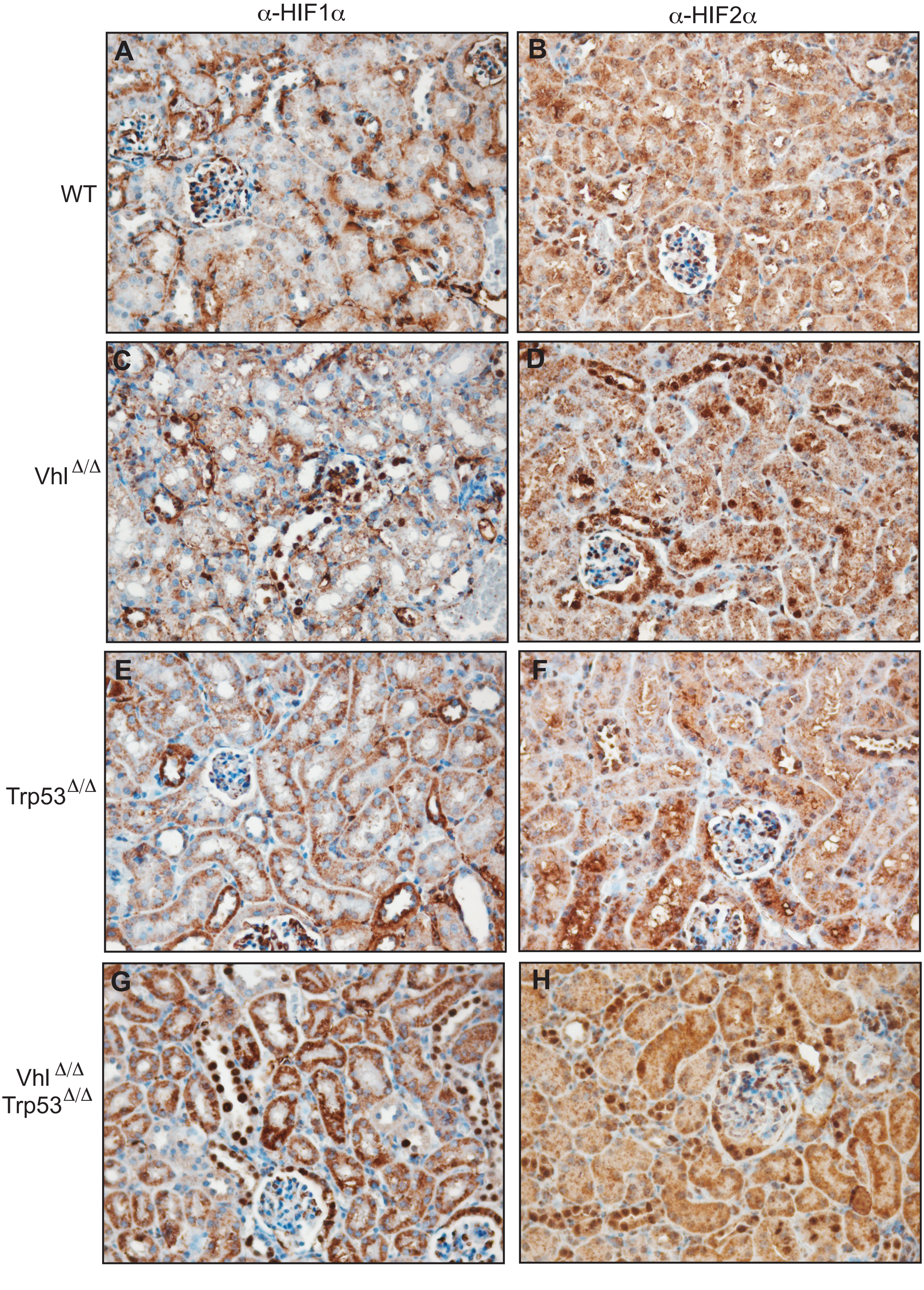

Supporting Information Fig 2. *Trp53* deletion does not alter nuclear accumulation of Hif1α and Hif2α following *Vhl* deletion in kidney epithelia.
Anti-HIF1α (**A,C,E,G**) and anti-HIF2α (**B,D,F,H**) immunohistochemistry of kidney cortex from five month-old wild type (**A,B**), Vhlh$^{\Delta/\Delta}$ (**C,D**), Trp53$^{\Delta/\Delta}$ (**E,F**) and Vhlh$^{\Delta/\Delta}$Trp53$^{\Delta/\Delta}$ (**G,H**) mice.

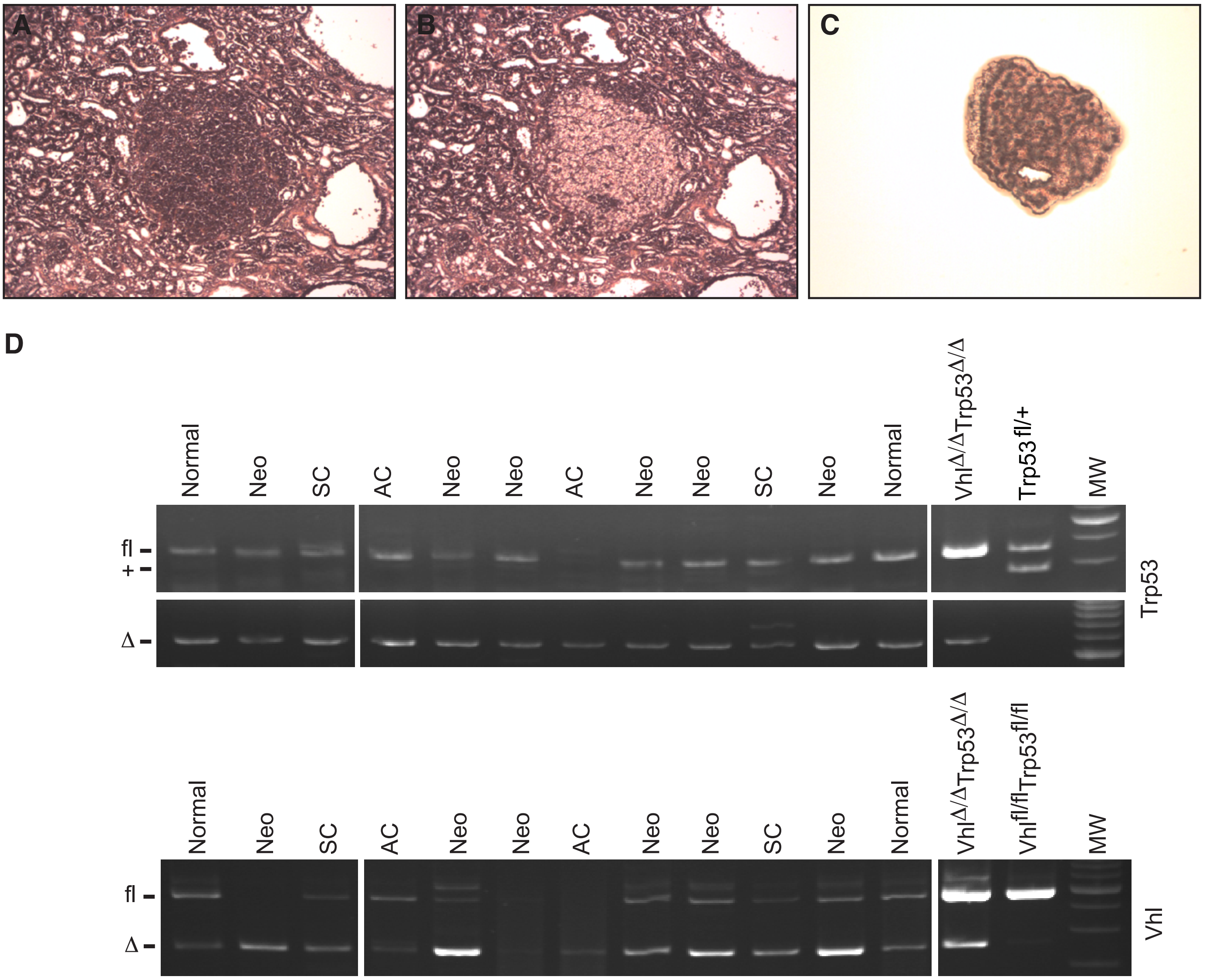

Supporting Information Fig 3. Analysis of recombination at the *Trp53* and *Vhl* loci in lesions from Vhl$^{\Delta/\Delta}$Trp53$^{\Delta/\Delta}$ mice.

A-C, Exmple of a neoplasm before (**A**) and after (**B**) laser capture microdissection and the captured tumour sample (**C**). Microdissection was performed with the Arcturus system using Capsure Macro LCM Caps (Applied Biosystems) and DNA was prepared using the Picopure DNA extraction kit (Applied Biosystems).

D, PCR genotyping analyses for recombination at the *Trp53* floxed and *Vhl* floxed loci using DNA extracted from microdissected histologically normal tissue (Normal), simple cysts (SC), atypical cysts (AC) or neoplastic lesions (Neo) from kidneys of Vhl$^{\Delta/\Delta}$Trp53$^{\Delta/\Delta}$ mice. DNA isolated from an entire section of a kidney from a Cre-expressing Vhl$^{\Delta/\Delta}$Trp53$^{\Delta/\Delta}$ mouse or from non-Cre-expressing Trp53$^{fl/fl}$Vhl$^{fl/fl}$ or Trp53$^{fl/+}$ mice served as controls for the PCRs. Positions of the floxed (fl), wild type (+) and recombined (Δ) *Trp53* and *Vhl* alleles are indicated.

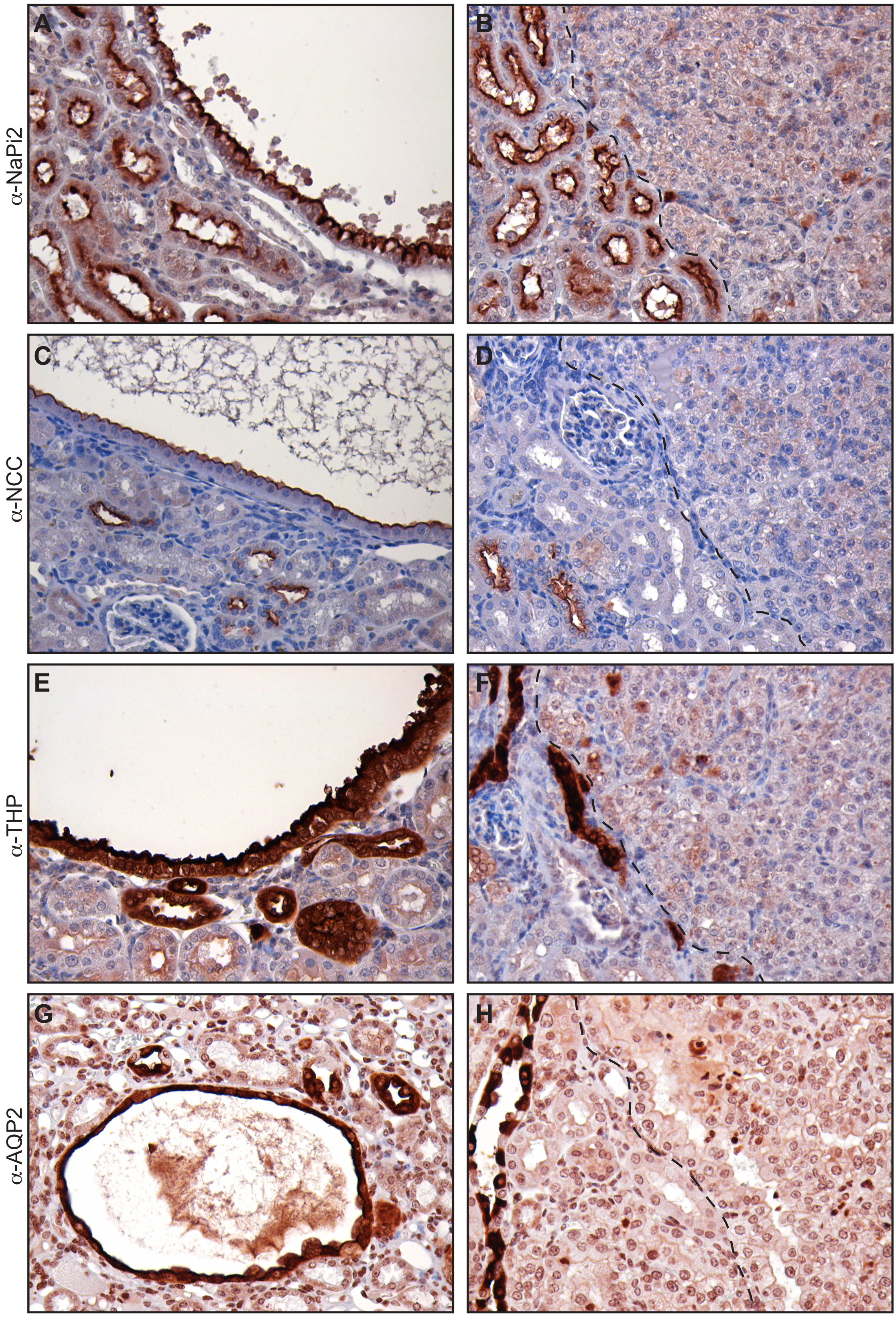

Supporting Information Fig 4. Simple cysts but not neoplasms express markers of specific tubular segments.

A,C,E,G, Examples of simple cysts arising in $Vhl^{\Delta/\Delta}Trp53^{\Delta/\Delta}$ mice that stain positively for NaPi2 (**A**), NCC (**C**), THP (**E**) and AQP2 (**G**) marking proximal tubules, distal convoluted tubules, thick ascending loops of Henle and collecting ducts, respectively.

B,D,F,H, Example of a neoplasm that does not stain positively for NaPi2 (**B**), NCC (**D**), THP (**F**) or AQP2 (**H**). Dotted lines illustrate the boundary between normal tissue (left of the line) and the neoplasm (right of the line).

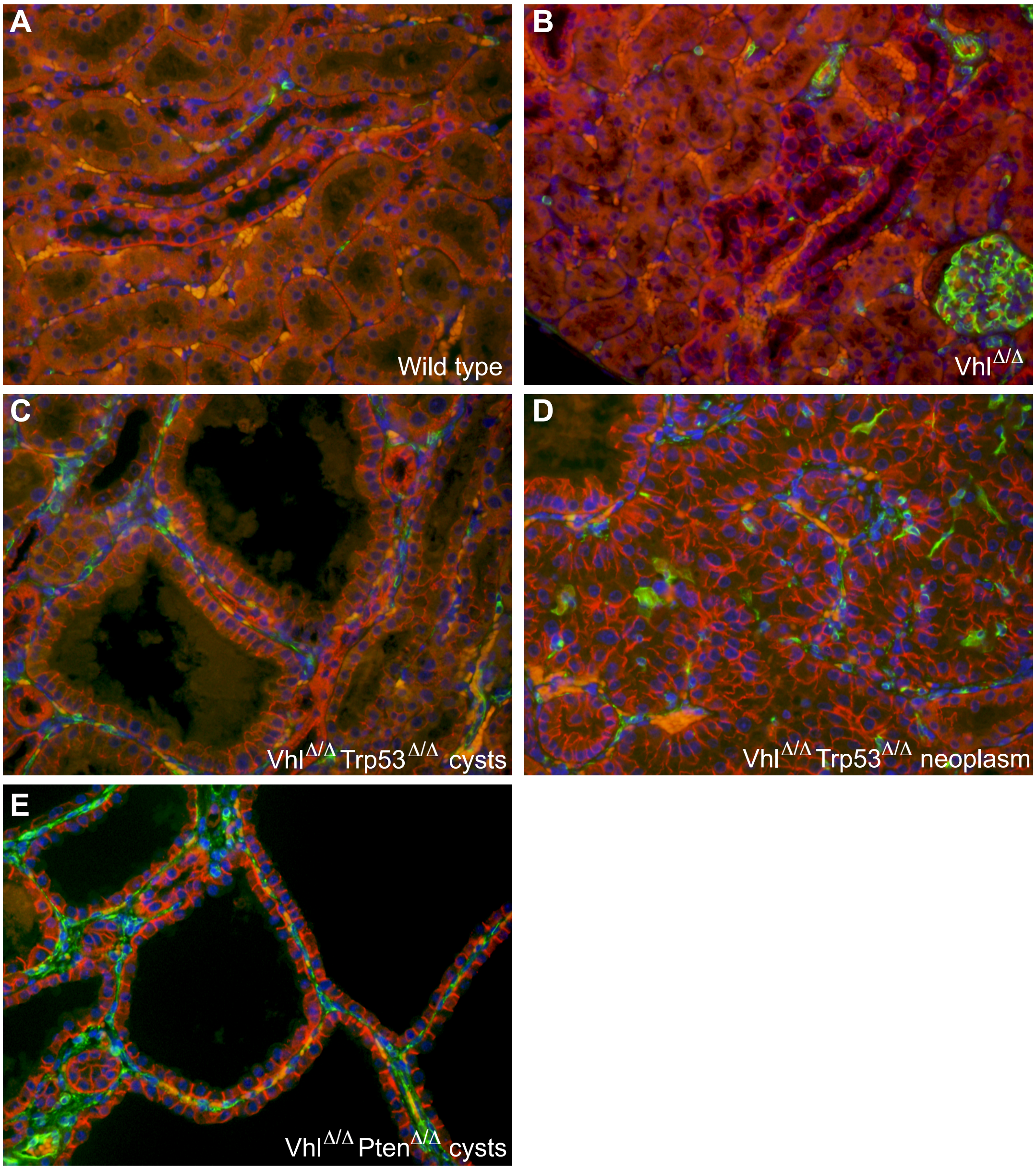

Supporting Information Fig 5. Lack of epithelial to mesenchymal transition following deletion of *Vhl* in mouse kidney epithelia, cysts and neoplasms.

A-E, Immunofluorescence staining for the epithelial marker E-cadherin (red), the mesenchymal marker vimentin (green) and DAPI (blue) to mark nuclei in a section from the cortex of a kidney from a wild type (**A**) or Vhl$^{\Delta/\Delta}$ (**B**) mouse, simple cysts (**C**) or a neoplasm (**D**) from a Vhl $^{\Delta/\Delta}$Trp53$^{\Delta/\Delta}$ mouse, or simple cysts from a Vhl$^{\Delta/\Delta}$Pten$^{\Delta/\Delta}$ mouse. Note the strong staining for E-cadherin and absence of vimentin staining in cystic and neoplastic cells. Vimentin expression is restricted to glomeruli, blood vessels and fibroblasts.

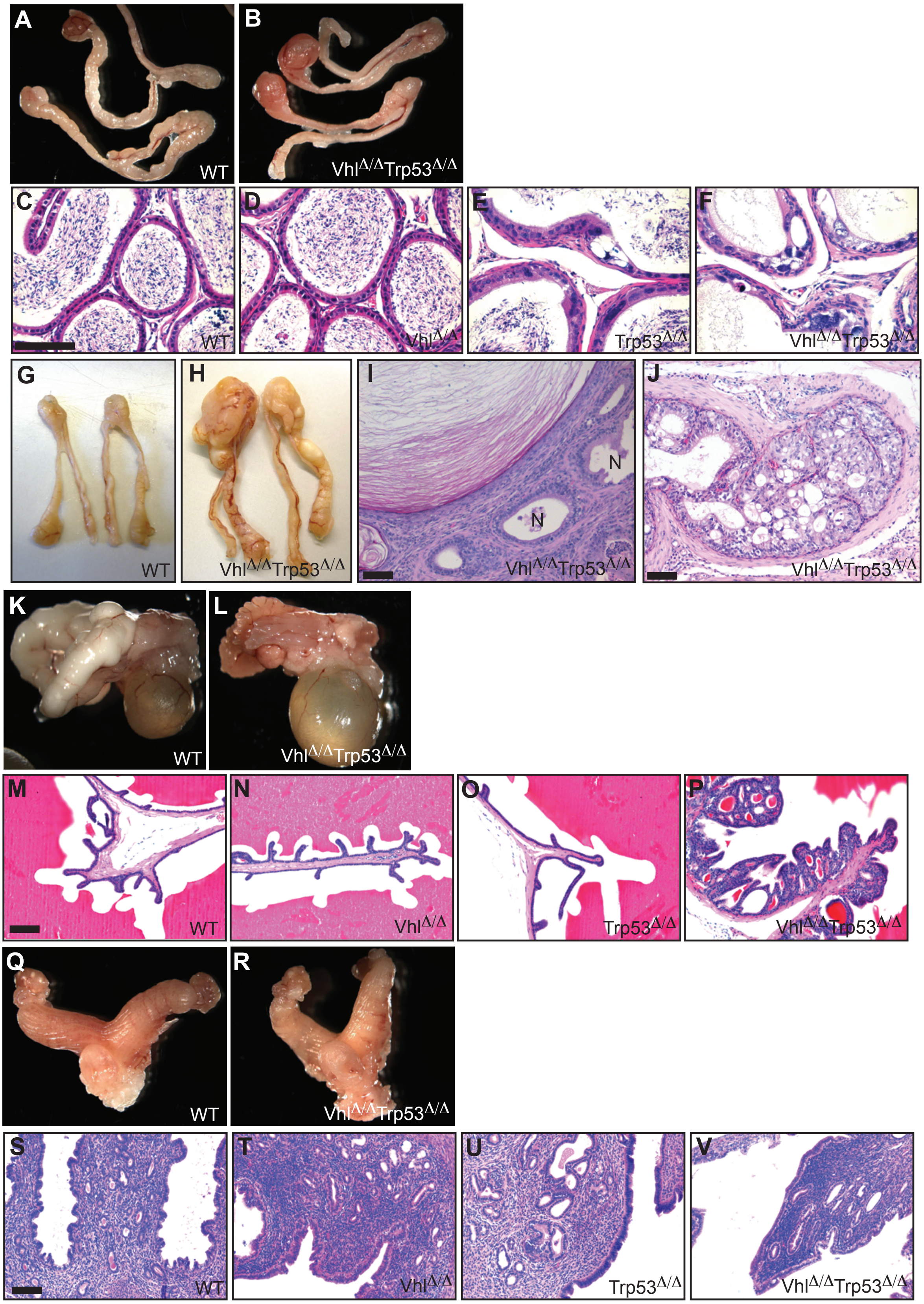

Supporting Information Fig 6. Vhlh$^{\Delta/\Delta}$Trp53$^{\Delta/\Delta}$ mice show multiple genital tract abnormalities

A-J, Epididymal phenotypes of Vhlh$^{\Delta/\Delta}$Trp53$^{\Delta/\Delta}$ mice. External appearance of epididymides from 2 month-old wild type (**A**) and Vhlh$^{\Delta/\Delta}$Trp53$^{\Delta/\Delta}$ (**B**) mice. Histological appearance of cauda regions of epididymides from 6 month-old wild type (**C**), Vhlh$^{\Delta/\Delta}$ (**D**), Trp53$^{\Delta/\Delta}$ (**E**) and Vhlh$^{\Delta/\Delta}$Trp53$^{\Delta/\Delta}$ (**F**) mice showing disorganisation of epithelial structure and frequent aberrant nuclear appearance in Trp53$^{\Delta/\Delta}$ and Vhlh$^{\Delta/\Delta}$Trp53$^{\Delta/\Delta}$ mice. External appearance of epididymides from 1 year-old wild type (**G**) and Vhlh$^{\Delta/\Delta}$Trp53 $^{\Delta/\Delta}$ (**H**) mice. Regions of squamous metaplasia (**I**) and extensive cribiform intraductal proliferation (**J**) were evident the majority of 1 year-old Vhlh$^{\Delta/\Delta}$Trp53$^{\Delta/\Delta}$ mice.

K-P, Vesicular gland phenotypes of Vhlh$^{\Delta/\Delta}$Trp53$^{\Delta/\Delta}$ mice. External appearance of vesicular glands from 2 month-old wild type (**K**) and Vhlh$^{\Delta/\Delta}$Trp53$^{\Delta/\Delta}$ (**L**) mice displaying the abberant formation of this structure. Histological appearance of epithelia of vesicular glands of 6 month-old wild type (**M**), Vhlh$^{\Delta/\Delta}$ (**N**), Trp53$^{\Delta/\Delta}$ (**O**) and Vhlh$^{\Delta/\Delta}$Trp53$^{\Delta/\Delta}$ (**P**) mice showing disorganised, multilayered, glandular structures of epithelia in Vhlh$^{\Delta/\Delta}$Trp53$^{\Delta/\Delta}$ mice.

Q-V, Analysis of uteri of Vhlh$^{\Delta/\Delta}$Trp53$^{\Delta/\Delta}$ mice. External appearance of uteri of 2 month-old wild type (**Q**) and Vhlh$^{\Delta/\Delta}$Trp53$^{\Delta/\Delta}$ (**R**) mice. Histological appearance of lumenal and glandular endometrial epithelia 6 month-old wild type (**S**), Vhlh$^{\Delta/\Delta}$ (**T**), Trp53$^{\Delta/\Delta}$ (**U**) and Vhlh$^{\Delta/\Delta}$Trp53$^{\Delta/\Delta}$ (**V**) mice showing no obvious defects in these tissues in Vhlh$^{\Delta/\Delta}$Trp53$^{\Delta/\Delta}$ mice. (**C-F**) are the same magnification, (**M-P**) are the same magnification, (**S-V**) are the same magnification. All scale bars depict 100μm.

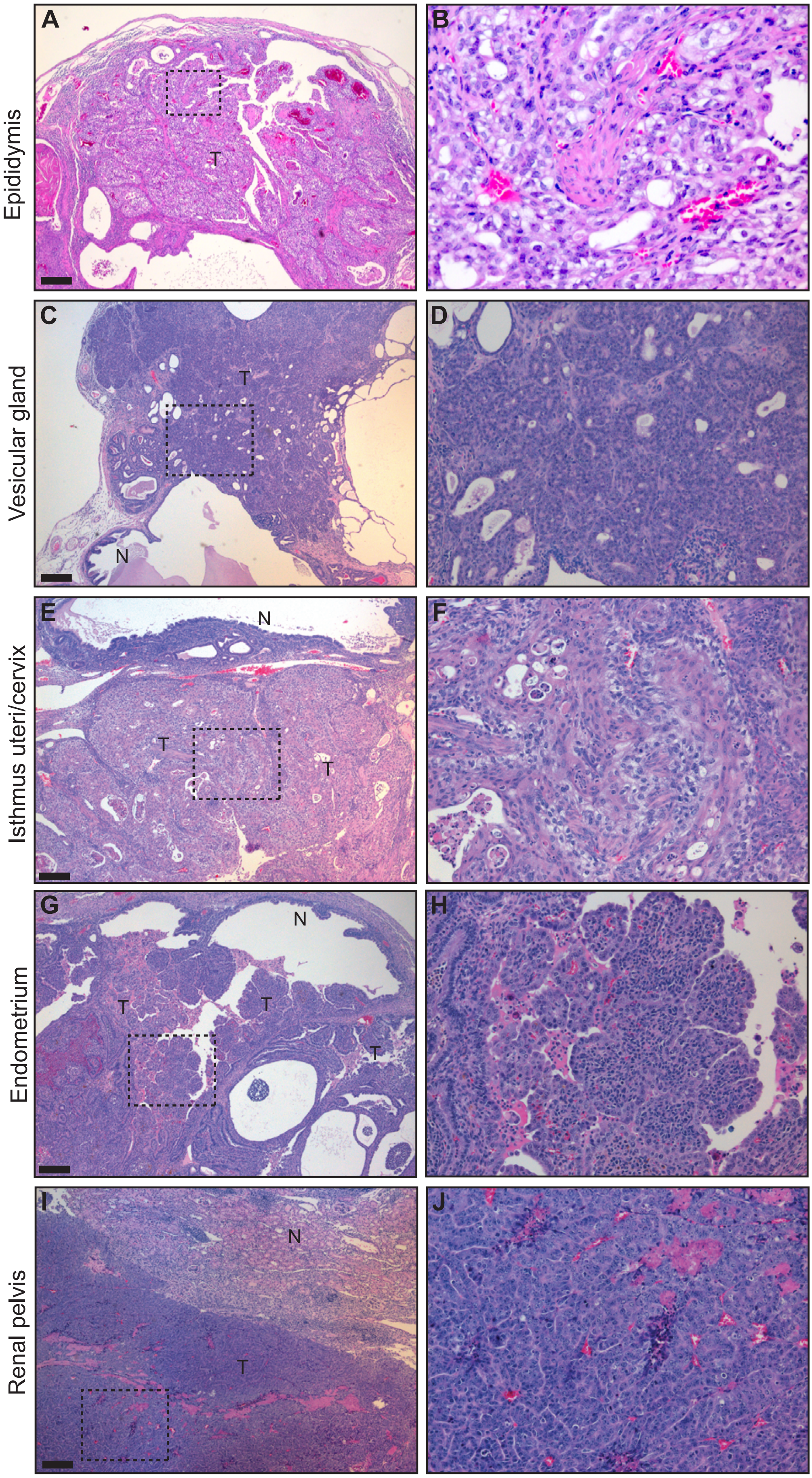

Supporting Information Fig 7. Tumours arising in one year-old Vhlh$^{\Delta/\Delta}$Trp53$^{\Delta/\Delta}$ mice.
A, Epididymal clear cell papillary cystadenoma. **C,** Carcinomas of the vesicular gland were seen in two mice. **E,** Squamous cell carcinoma in isthmus uteri, likely upper cervical origin. **G,** High grade endometrial adenocarcinoma. **I,** Poorly differentiated, high grade metastatic carcinoma, likely origin in the urothelium of the renal pelvis. B,D,F,H,J are zooms of the boxed regions in A,C,E,G,I respectively. Scale bars in A,C,E,G,I depict 200 µm. N: normal tissue, T: tumour.

Supporting Information Table 1. Primers used for *TP53* PCR and sequence analysis.

**Primer**	**Sequence**
TP53 Exon 5 forward	5'-CACTTGTGCCCTGACTTTCA-3'
TP53 Exon 5 reverse	5'-AACCAGCCCTGTCGTCTCT-3'
TP53 Exon 6 forward	5'-CAGGCCTCTGATTCCTCACT-3'
TP53 Exon 6 reverse	5'-CTTAACCCCTCCTCCCAGAG-3'
TP53 Exon 7 forward	5'-CCACAGGTCTCCCCAAGG-3'
TP53 Exon 7 reverse	5'-CAGCAGGCCAGTGTGCAG-3'
TP53 Exon 8 forward	5'-GCCTCTTGCTTCTCTTTTCC-3'
TP53 Exon 8 reverse	5'-TAACTGCACCCTTGGTCTCC-3'
